# Activity–Selectivity of Flavonoid Derivatives in Endometriotic Cells

**DOI:** 10.1021/acsomega.5c12546

**Published:** 2026-02-02

**Authors:** Kaio S. Gomes, Julia A. Coelho, Pedro E. H. Tesser, Dalete C. S. Souza, Matheus L. Silva, Edgard A. Ferreira, Joao H. G. Lago, Giselle Cerchiaro

**Affiliations:** 1 Metal Biochemistry and Oxidative Stress Laboratory, Centre for Natural Sciences and Humanities, Federal University of ABC, Santo Andre, SP 09210-5800, Brazil; 2 Laboratory of Chemical Biology, Centre for Natural Sciences and Humanities, Federal University of ABC, Santo Andre, SP 09210-580, Brazil; 3 School of Engineering, Mackenzie Presbyterian University, São Paulo, SP 01302-907, Brazil

## Abstract

Natural polyphenolics, more specifically flavonoids and derivatives, constitute chemically versatile scaffolds with a broad biological potential. In this study, different flavonoid derivatives (**1**–**37**) were assessed for cytotoxicity in Ishikawa and 12Z epithelial cell lines, serving as models of eutopic endometrium and endometriosis, respectively, to elucidate structure–activity relationships. Flavonoids bearing multiple hydroxyl and methoxy substituents exhibited high polarity, an elevated topological polar surface area (TPSA), and numerous hydrogen-bond donors and acceptors, consistently demonstrating low cytotoxicity and, in several cases, cytoprotective effects. In contrast, chalcones containing electron-withdrawing substituents (−NO_2_ and −Cl) and higher lipophilicity (log *P* > 3.5) displayed marked and selective toxicity toward 12Z cells. Among these, compounds **24** [(*E*)-3-(4-(dimethylamino)­phenyl)­1-(3-hydroxyphenyl)­prop-2-en-1-one] and **28** [(*E*)-3-(benzo­[*d*]­[1,3]­dioxol-5-yl)-1-(4-chlorophenyl)­prop-2-en-1-one)] emerged as the most promising selective candidates, reducing 12Z cell viability to approximately 50% while maintaining or enhancing Ishikawa cell viability (>100%). Additional derivatives, including **14** [(*E*)-3-(benzo­[*d*]­[1,3]­dioxol-5-yl)-1-phenylprop-2-en-1-one], **17** [(*E*)-3-(benzo­[*d*]­[1,3]­dioxol-5-yl)-1-(4-nitrophenyl)­prop-2-en-1-one], **23** [(*E*)-3-(4-(dimethylamino)­phenyl)-1-(2-hydroxyphenyl)­prop-2-en-1-one], and **30** [(*E*)-1-phenyl-3-(3,4,5-trimethoxyphenyl)­prop-2-en-1-one], also exhibited statistically significant selectivity. Correlation analysis further revealed a strong association between lipophilicity and 12Z cytotoxicity (*r* = −0.73), whereas elevated TPSA and extensive hydrogen bonding correlated with cytoprotective behavior. Collectively, these results highlight chalcones as promising molecular frameworks in which substituent-dependent physicochemical properties are associated with distinct biological outcomes, ranging from selective endometriotic cytotoxicity to endometrial cytoprotective effects.

## Graphical abstract

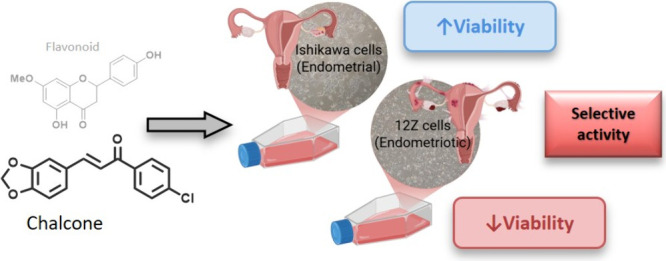

## Highlights

- Different flavonoid derivatives (**1**–**37**) were evaluated for cytotoxicity in endometrial (Ishikawa) and endometriotic (12Z) cell models.
- Flavonoids with hydroxyl/methoxy substituents, high TPSA, and multiple H-bond donors/acceptors showed low cytotoxicity and cytoprotective effects.
- Flavonoids carrying electron-withdrawing substituents (−NO_2_ and −Cl) and increased lipophilicity (log *P* > 3.5) displayed selective toxicity toward 12Z cells.
- Compounds **24** [(*E*)-3-(4-(dimethylamino)­phenyl)­1-(3-hydroxyphenyl)­prop-2-en-1-one] and **28** [(*E*)-3-(benzo­[*d*]­[1,3]­dioxol-5-yl)-1-(4-chlorophenyl)­prop-2-en-1-one] emerged as the most selective agents, reducing 12Z viability to ∼50% while preserving or enhancing Ishikawa viability.
- Structure–activity relationships suggested that lipophilicity contributes to selective toxicity, positioning chalcones as promising candidates for endometriosis therapy.

## 1 Introduction

Endometriosis is a benign gynecological disorder with a multifactorial etiology involving genetic, epigenetic, immunological, hormonal, and environmental factors. These mechanisms collectively contribute to chronic inflammation and oxidative stress arising from the presence of ectopic endometrial tissues. The disease can be classified into three major forms: endometrioma, deep endometriosis, and superficial peritoneal endometriosis.^1,2^ The incidence of endometriosis affects up to 20% of women of reproductive age, with common clinical manifestations including dysmenorrhea, chronic pelvic pain, abnormal bleeding, and infertility.^3–6^

Current therapeutic approaches typically combine laparoscopic surgery with hormonal treatments that aim at lesion removal and pain relief. However, these strategies often fail to restore fertility or prevent disease recurrence. Surgical intervention is associated with recurrence rates of approximately 20%,^7^ while hormonal manipulation frequently induces adverse effects due to steroid hormone imbalance.^2,4^ Given the significant impact of endometriosis on the quality of life and the limitations of existing treatments, which primarily target symptom control rather than the underlying pathophysiology, there is a critical need for the development of new therapeutic agents to improve disease management.

Natural products constitute an invaluable source of therapeutic agents, owing to their remarkable structural diversity. According to Newman and Cragg (2020), approximately 48% of all new drugs approved by the FDA between 1981 and 2019 were derived from natural sources.^8^ Among these, phenolic compounds represent a highly diverse group of plant metabolites recognized for their broad biological potential, particularly their antioxidant, anti-inflammatory, and anticancer activities.^9–17^

Flavonoids, in particular, are capable of scavenging free radicals and reducing oxidative stress, while modulating the activity of several enzymes and signaling pathways, thereby reinforcing their protective effects on human health.^18–26^ Due to their versatile reactivity, chalcones have emerged as privileged chemical scaffolds, serving as key intermediates in the design of a wide range of biologically active derivatives exhibiting anti-inflammatory, antihistaminic, antioxidant, antiobesity, antiparasitic, and other pharmacological properties.^15,27–32^

Despite these advances, experimental studies addressing endometriosis at the cellular level remain limited, while a substantial body of recent research has focused on patient-based and clinical investigations.^1,7^ Most in vitro investigations have focused on inflammatory signaling, oxidative stress, or hormonal responsiveness, frequently relying on single-cell-line systems or reporting general cytotoxic effects without direct comparison between endometriotic and eutopic endometrial cells. Importantly, a small number of established cellular models continue to underpin most experimental work in this area, and comparative screening approaches have changed little over time. As a result, systematic evaluations of differential cytotoxic responses and selectivity in endometriosis-relevant cellular models are still scarce.^33,34^

In this context, the present study aimed to evaluate the cytotoxic and selective properties of a series of different flavonoid derivatives (**1**–**37**) as an initial screening step, using models of normal endometrial (Ishikawa) and endometriotic (12Z) cells.^33,34^ The investigation focused on correlating key physicochemical descriptors, such as lipophilicity (log *P*), topological polar surface area (TPSA), and hydrogen-bonding capacity, with the observed biological responses to identify patterns associated with cytoprotective versus cytotoxic selectivity.

## 2 Results and Discussion

A set of flavonoid derivatives (**1**–**37**, Figure 1) had their cytotoxicity evaluated for the Ishikawa cell line, commonly used to simulate the normal endometrium, by the MTT assay.

**Figure 1:**
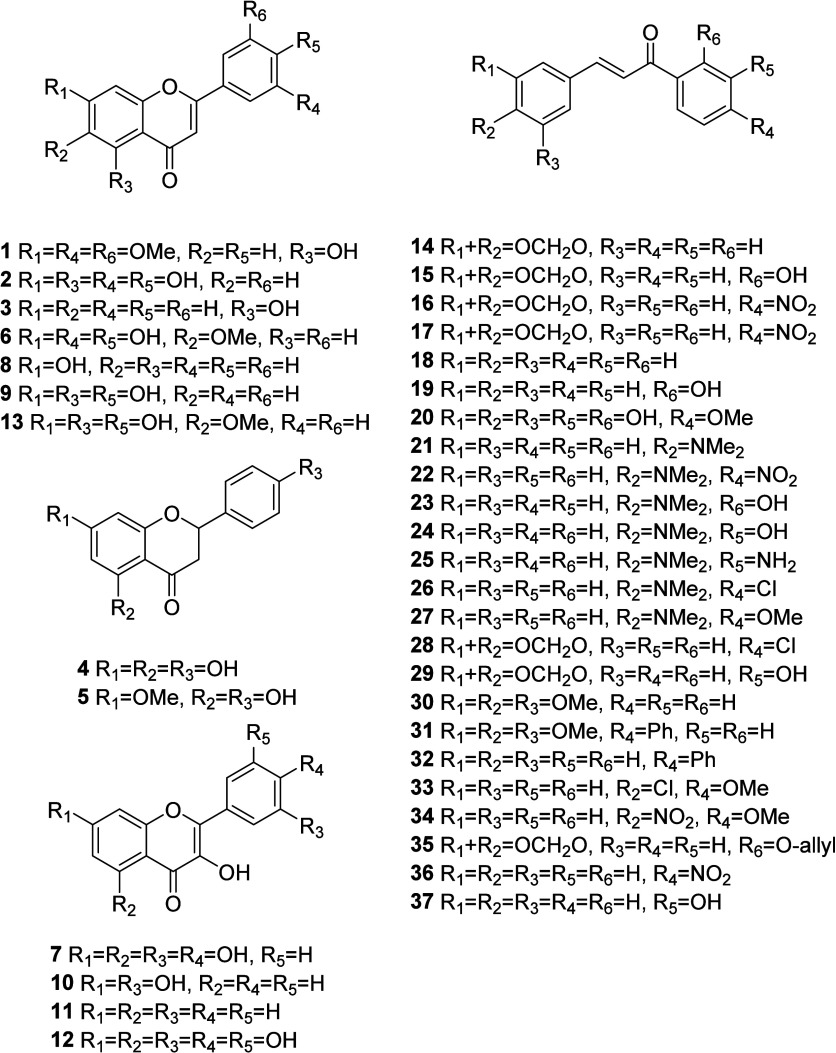
Structures of flavonoid derivatives **1**–**37** evaluated for cytotoxicity in Ishikawa and 12Z cell lines.

The viability assay conducted in Ishikawa cells (Figure 2) revealed a broad spectrum of biological responses across the compound library. A subset of molecules exhibited cytoprotective activity, with viabilities exceeding 100%, including **1** (155%), **2** (150%), **6** (102%), **7** (149%), **8** (126%), **17** (127%), **28** (114%), and **30** (114%). These elevated viability values may reflect enhanced epithelial cell stratification or increased mitochondrial activity, as detected by the MTT assay employed. Considering chemical aspects, several compounds correspond to polyhydroxylated or methoxylated flavonoids (e.g., **1**, **2**, and **7**), whereas others possess fewer polar substituents (**8**) or halogen groups (**28**). This result suggests that cytoprotective effects are not solely determined by the number of hydroxyl or methoxyl groups but rather by the nature, position, and overall electronic interplay of substituents within the scaffold. A second group of molecules displayed nontoxic behavior, maintaining viabilities between 75% and 100%, including **5** (83%), **13** (87%), **14** (82%), **15** (78%), **23** (84%), and **26** (81%). These derivatives preserved near-baseline cell viability without a consistent substitution pattern, indicating that no single substituent class could be identified as a dominant determinant of cytoprotective behavior within the present data set.

**Figure 2:**
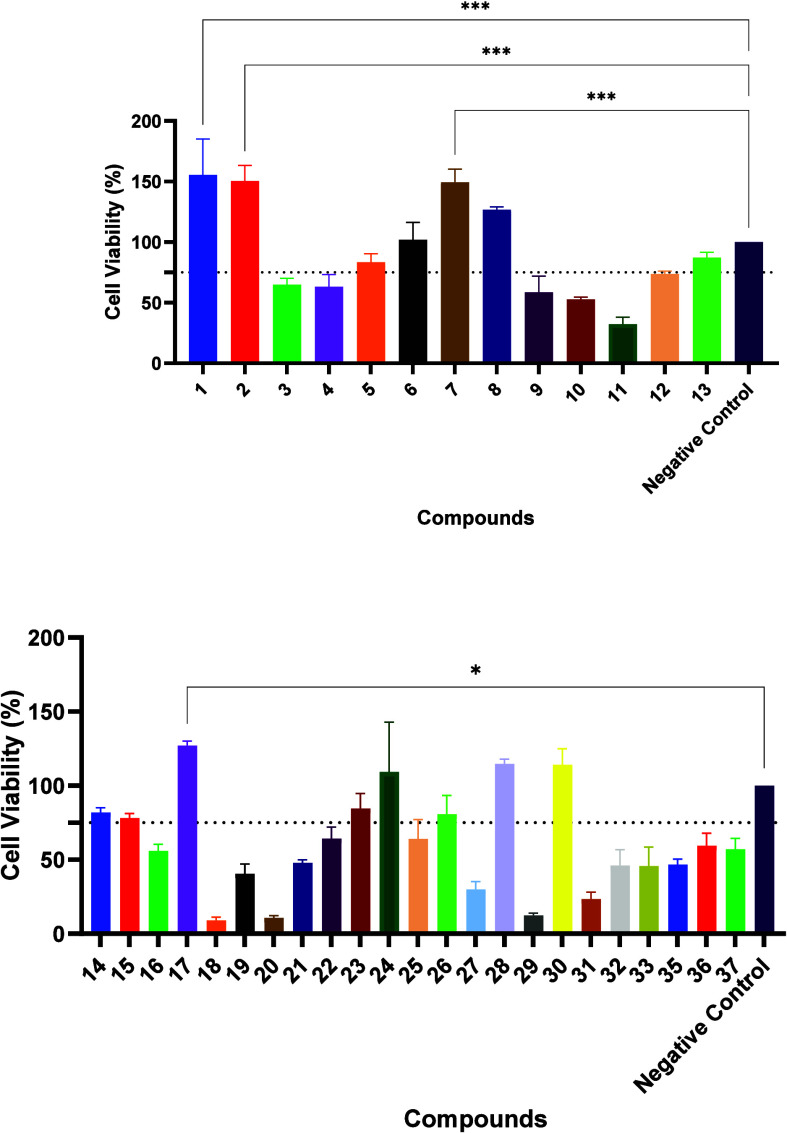
Cell viability of Ishikawa cells after exposure to compounds **1**–**37** at 200 μM for 24 h. Data are expressed as means ± SD relative to the negative control (untreated cells, 100%). Viability was determined by the MTT assay. *N* = 3 independent experiments performed in triplicate. Statistical significance versus the negative control was assessed by one-way ANOVA with Bonferroni’s post-test, with significance represented as *p* < 0.05 (*) and *p* < 0.001 (***).

Conversely, several compounds exhibited cytotoxicity toward Ishikawa cells such as **3** (65%), **4** (63%), **9** (58%), **10** (53%), **11** (32%), and **29** (12%). This group included compound **18**, the only unsubstituted chalcone in the series, as well as derivatives containing electron-withdrawing substituents such as −NO_2_ (**16**, **22**, **34**, and **36**) or −Cl groups (**25** and **33**). Other compounds, including **29**, **31**, **32**, and **37**, also showed high cytotoxicity, possibly due to the absence of polar donor substituents. Having established an initial safety profile in Ishikawa cells, subsequent assays were performed to evaluate the activity in 12Z cells.

In 12Z cells, several compounds exhibited pronounced inhibitory effects (Figure 3), with the greatest reductions in viability observed for **24** (49%), **15** (51%), **28** (53%), **23** (59%), and **14** (60%). These derivatives, which reduced the viability to approximately 60% or below, stand out as promising lead candidates for targeting endometriotic cells. A second group demonstrated moderate inhibition, maintaining viability between 70 and 90%, such as **30** (70%), **17** (74%), **5** (82%), **6** (86%), and **8** (88%). Although less potent, these compounds remain noteworthy when considering their fixed-dose selectivity, defined here by comparing 12Z and Ishikawa cell viability under identical exposure conditions.

**Figure 3:**
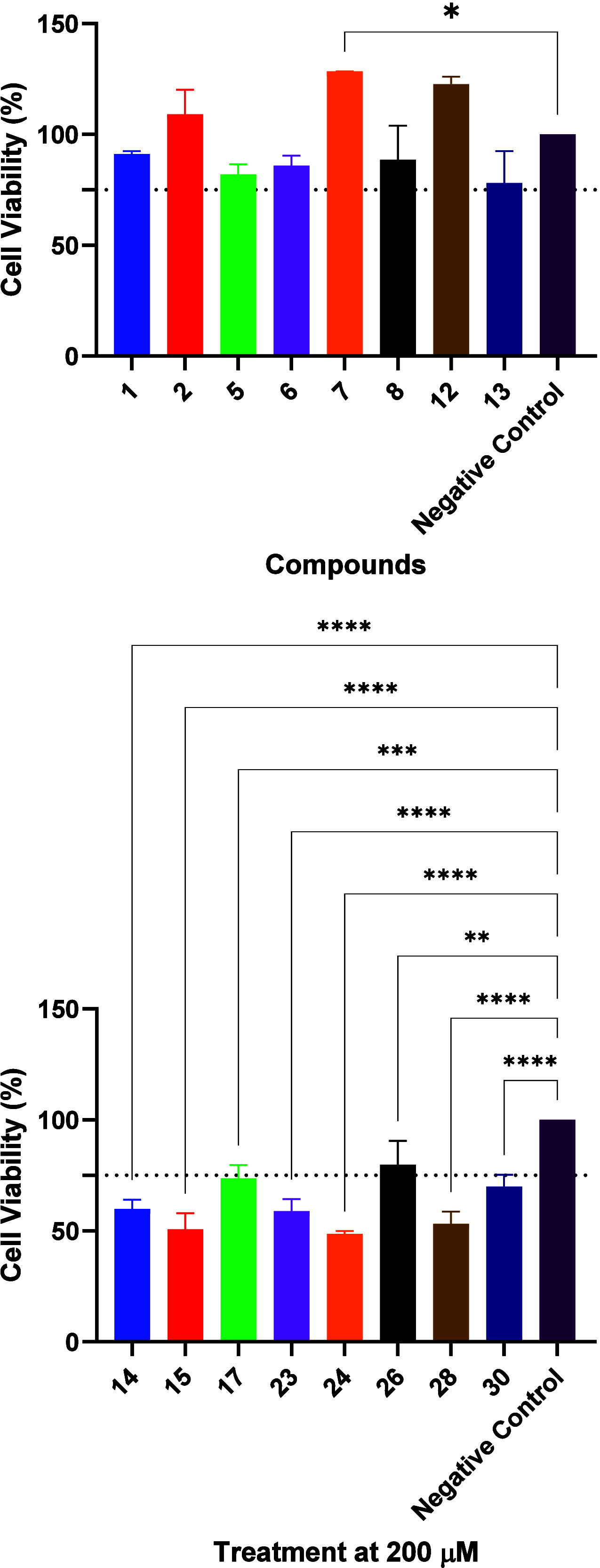
Cell viability of 12Z cells after exposure to compounds **1**, **2**, **5**–**8**, **12**–**15**, **17**, **23**, **24**, **26**, **28**, and **30** at 200 μM for 24 h. Data are expressed as means ± SD relative to untreated cells (100%). Viability was determined by the MTT assay. *N* = 3 independent experiments performed in triplicate. Statistical significance versus the control was assessed by one-way ANOVA with Bonferroni’s post-test with significance represented as *p* < 0.05 (*), *p* < 0.01 (**), *p* < 0.001 (***), and *p* < 0.0001 (****).

In contrast, certain compounds produced cytoprotective or neutral effects in 12Z cells, with viability values exceeding 100%, indicating undesirable selectivity in this model, as they may promote the survival of diseased cells. Overall, the 12Z data identify compounds **14**, **15**, **23**, **24**, and **28** as the most effective inhibitors of endometriotic cell viability, while also highlighting a subset of derivatives that are inactive or potentially counterproductive due to their prosurvival effects.

Selectivity between the two cell lines was evaluated by directly comparing the viability at a fixed concentration (200 μM, 24 h). The underlying rationale was that ideal candidates should effectively reduce 12Z viability while sparing Ishikawa cells, thereby combining potency with safety. Statistical analysis revealed that compounds **1**, **2**, **14**, **17**, **23**, **24**, **28**, and **30** exhibited significant differences between the two cell lines. Among these, **24** (49% in 12Z vs 109% in Ishikawa, *p* < 0.001) and **28** (53% vs 115%, *p* < 0.0001) demonstrated the highest selectivity, markedly reducing 12Z viability while preserving or even enhancing Ishikawa viability, representing cytotoxic selectivity (preferential 12Z cell death).

Compounds **14**, **17**, **23**, and **30** also showed statistically supported selectivity, albeit with smaller margins, maintaining Ishikawa viability above 80% while reducing 12Z viability into the 50–70% range. Conversely, compounds **1** and **2**, although displaying statistically significant differences (*p* < 0.0001 and *p* = 0.0101, respectively), retained high 12Z viability (>90%), indicating cytoprotective selectivity (preferential protection of Ishikawa rather than elimination of 12Z). A secondary group, including compounds **6**–**8** and **15**, exhibited a trend toward selectivity, showing consistent but statistically nonsignificant differences between the two cell lines.

Importantly, these results highlight two biologically distinct patterns of selectivity at this fixed concentration: (i) preferential impairment of 12Z viability, the desired therapeutic profile, as observed for compounds **24** and **28**; and (ii) preferential protection of Ishikawa cells, a context-dependent and less therapeutically favorable pattern, as seen for compounds **1** and **7**. Collectively, these findings identify compounds **24** and **28** as the most promising selective agents, with **14**, **17**, **23**, and **30** also emerging as statistically validated hits.

Although evaluated at a single concentration, this analysis provides a robust framework for assessing therapeutic selectivity: compounds that reduce 12Z viability while maintaining Ishikawa viability are considered to be the most promising candidates. Table 1 summarizes the viability data for both cell lines alongside calculated physicochemical descriptors (log *P*, TPSA, HBD, and HBA), providing the data set underlying the selectivity assessment (Figure 4) and subsequent structure–activity relationship analysis.

**Figure 4:**
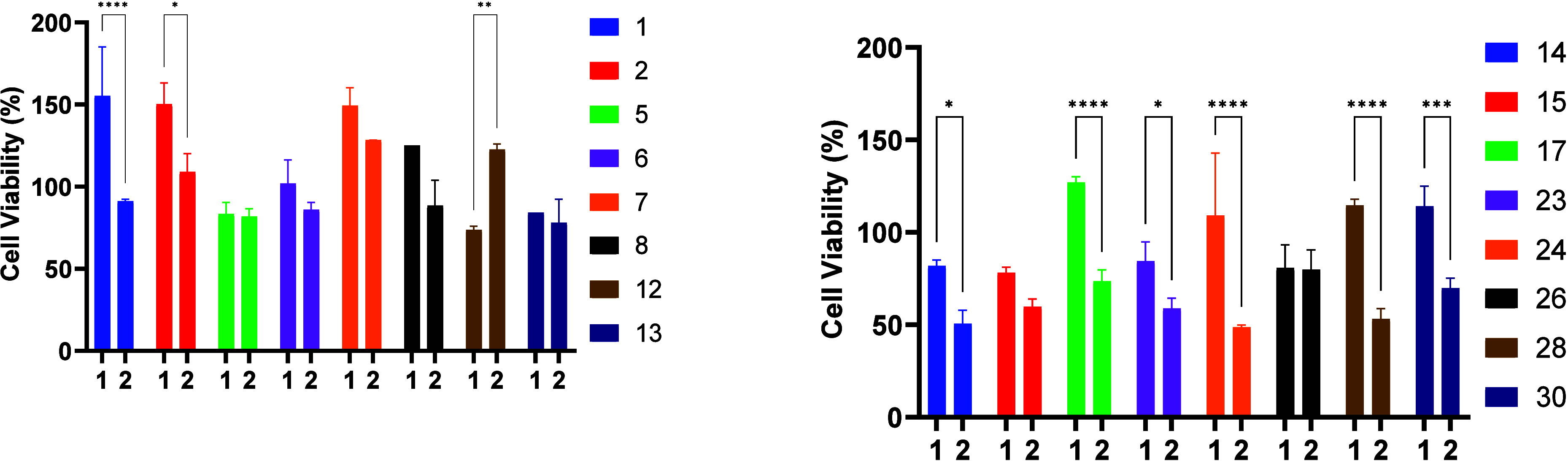
Comparison of cell viability between Ishikawa, indicated as **1**, and 12Z, indicated as **2**, cells after exposure to selected compounds (**1**, **2**, **5**–**8**, **12**–**15**, **17**, **23**, **24**, **26**, **28**, and **30**) at 200 μM for 24 h. Data are expressed as means ± SD relative to untreated cells (100%). Statistical analysis was performed using two-way ANOVA with Bonferroni’s post-test with *p* < 0.05 (*), *p* < 0.01 (**), *p* < 0.001 (***), and *p* < 0.0001 (****).

**Table 1:** Viability of Ishikawa and 12Z Cells after Treatment with Selected Compounds (200 μM, 24 h) Expressed as Means ± SD Relative to Untreated Cells (100%)^a^

	cell viability (% ± SD)						
compound	Ishikawa	12Z	Δviability	log *P*	**TPSA (Å** **^2^** **)**	HBD	HBA
1	155.36 ± 29.73	91.19 ± 1.20	64.17	3.19	78.13	1	6
2	150.36 ± 12.81	109.02 ± 11.12	41.34	2.28	111.13	4	6
17	127.05 ± 3.05	73.63 ± 6.06	53.42	3.22	78.67	0	5
23	84.51 ± 10.28	58.93 ± 5.40	25.58	3.35	40.54	1	3
24	109.23 ± 33.65	48.65 ± 1.29	60.58	3.35	40.54	1	3
28	114.67 ± 3.33	53.18 ± 5.51	61.49	3.96	35.53	0	3
30	114.07 ± 10.87	69.91 ± 5.40	44.16	3.61	44.76	0	4

a Δviability represents the difference between Ishikawa and 12Z viability. Physicochemical descriptors (log *P*, TPSA, HBD, and HBA) were calculated using RDKit. *N* = 3 independent experiments performed in triplicate.

Cluster-based analysis was used as a descriptive tool to group compounds according to similarity in their physicochemical descriptors and observed biological responses (Figure 5). Cluster 1 comprised molecules bearing oxygenated substituents (−OH and −OMe) and nitro groups (−NO_2_), which displayed intermediate toxicity in both cell lines (compounds **14**, **15**, **16**, **17**, **28**, **29**, and **35**). This cluster reflects a recurring phenotypic response pattern within the investigated chemical space, rather than a definitive structure–activity relationship, and is associated with limited fixed-dose selectivity.^33^

**Figure 5:**
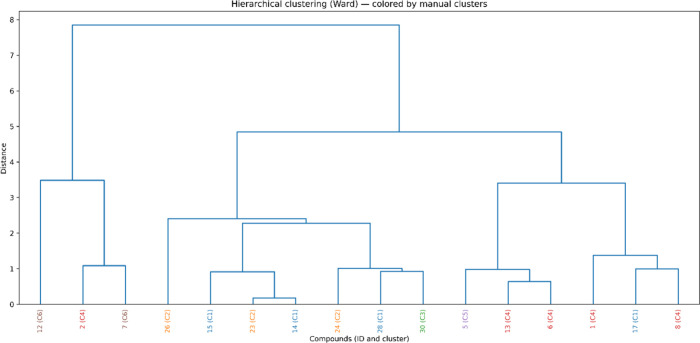
Structural clustering of the compound library. Hierarchical clustering (Ward method) separated the compounds into six clusters (C1–C6). Each cluster reflects recurring phenotypic response patterns observed at a fixed dose: C1–C2 grouped moderately active derivatives, C3 concentrated the most cytotoxic compounds (cell line-dependent), C4 and C6 contained polyhydroxylated/methoxylated structures associated with cytoprotective responses, and C5 comprised simpler molecules with intermediate viability.

Cluster 2, enriched with −NH_2_ and −NMe_2_ derivatives (compounds **21**–**27**), exhibited modest cytotoxicity and tended toward cytoprotective selectivity, maintaining high tolerability in both cell lines, typically with an Ishikawa cell viability of ≥80%. In contrast, cluster 3, composed of nitro- and halogen-substituted chalcones (compounds **18**–**20**, **30**–**34**, **36**, and **37**), included the most cytotoxic compounds toward Ishikawa cells. This cluster displayed cytotoxic-type selectivity when the 12Z viability was more strongly reduced than Ishikawa at the fixed dose.

Clusters 4 (compounds **1**, **2**, **3**, **6**, **8**, **9**, and **13**) and 6 (compounds **7**, **10**, **11**, and **12**), dominated by polyhydroxylated and methoxylated flavonoids, consistently maintained high viability (often >100%), corresponding to cytoprotective selectivity that is favorable for Ishikawa cells but undesirable for 12Z. Cluster 5 comprised flavanones (compounds **4** and **5**) featuring a partially saturated benzopyranone core, which displayed intermediate but largely nonselective biological profiles.

Overall, these findings indicate that selectivity within this compound set is best described by phenotypic response patterns associated with physicochemical properties, rather than by the scaffold type or mechanistic electronic interpretations.

Analysis of lipophilicity (log *P*), calculated from RDKit descriptors, revealed an empirical association between higher log *P* values and reduced cell viability, particularly in 12Z cells. Compounds in the upper quartile (log *P* > 3.5) were enriched among the more cytotoxic series, including members of clusters 1 and 3 (nitro- and halogen-substituted chalcones), which were among the compounds exhibiting stronger cytotoxic effects.^35,36^ In contrast, polyhydroxylated and methoxylated clusters (4 and 6) displayed significantly lower log *P* values (<3), corresponding to reduced cytotoxicity and, in some cases, proliferative effects consistent with polyphenol-associated cytoprotection.^37,38^ Within the fixed-dose selectivity framework, higher log *P* values were associated with a cytotoxic selectivity profile, characterized by preferential reduction of 12Z viability with preservation of Ishikawa cells, as exemplified by compounds **28** (log *P* = 3.96; 12Z ≈ 53%; Ishikawa ≈ 115%) and **24** (log *P* = 3.35; 12Z ≈ 49%; Ishikawa ≈ 109%). Conversely, lower log *P* values combined with multiple hydroxyl or methoxyl substituents (e.g., compound **1**, log *P* = 2.84) were associated with cytoprotective behavior, preserving Ishikawa viability while only modestly affecting 12Z cells.

The topological polar surface area (TPSA) was analyzed as an independent descriptor, with higher TPSA values associated with reduced cytotoxicity at the fixed dose.^39,40^ Compounds with TPSA > 80 Å^2^ (compounds **1**, **2**, **6**, **7**, **12**, and **13**), indicative of cytoprotective selectivity, are favorable for Ishikawa but undesirable for 12Z. In contrast, TPSA < 40 Å^2^ (compounds **28**, **29**, **32**, **33**, **34**, and **36**) was associated with greater cytotoxicity and, when the Ishikawa viability remained ≥80%, reflected cytotoxic-type selectivity. For example, compound **12** (TPSA ≈ 137 Å^2^) exhibited high polarity and cytoprotective bias (Ishikawa ≈ 70%; 12Z ≈ 120%), while compound **28**, with low TPSA and high log *P*, displayed preferential 12Z inhibition. Thus, under fixed-dose conditions, high TPSA favored cytoprotective selectivity, whereas low TPSA, particularly when accompanied by higher lipophilicity, was more frequently observed among compounds showing cytotoxic selectivity.

Hydrogen-bonding parameters, expressed as the number of hydrogen-bond donors (HBD) and acceptors (HBA), paralleled TPSA trends and further refined this interpretation. Polyhydroxylated derivatives (compounds **1**, **2**, **6**, **7**, **12**, and **13**), characterized by multiple hydrogen-bond donor and acceptor functionalities, consistently exhibited reduced cytotoxicity in both cell lines, frequently resulting in cytoprotective selectivity.^37,41,42^ In contrast, chalcones with fewer hydrogen-bonding features (compounds **28**, **29**, **32**–**34**, and **36**) displayed increased cytotoxicity and, when Ishikawa viability remained above 80%, demonstrated cytotoxic-type selectivity toward 12Z. Compound **12**, which presents multiple hydrogen-bonding functionalities, exemplified relative 12Z sparing compared with Ishikawa cells, whereas compound **28** (HBD = 0; HBA = 3) illustrated the opposite behavior, representing two extremes within the observed selectivity spectrum.

To integrate these relationships, a correlation heatmap was generated (Figure 6). log *P* correlated negatively with 12Z viability (*r* = −0.73), whereas TPSA, HBD, and HBA showed strong positive correlations (*r* = 0.77–0.83). Ishikawa viability exhibited only weak correlations (*r* ≤ 0.26). Importantly, Δviability (Ishikawa – 12Z) demonstrated weak, but consistent trends, negative with log *P* (*r* = −0.24) and positive with TPSA/HBD/HBA (*r* = 0.19–0.25), supporting the dual-mode selectivity model: higher lipophilicity drives cytotoxic selectivity toward 12Z, while higher polarity and hydrogen-bonding capacity favor cytoprotective selectivity toward Ishikawa.

**Figure 6:**
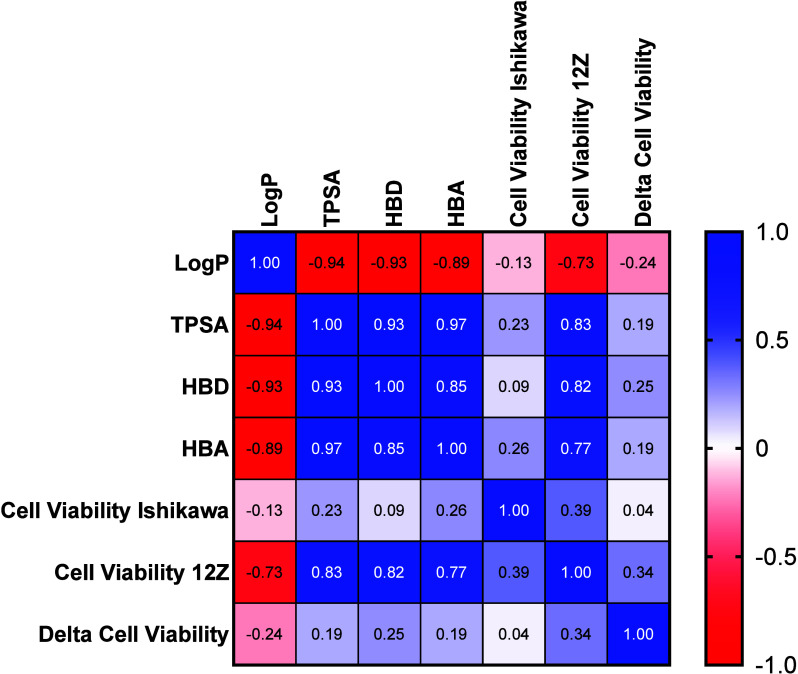
Correlation heatmap of physicochemical descriptors and biological outcomes for compounds **1**, **2**, **5**–**8**, **12**–**15**, **17**, **23**, **24**, **26**, **28**, and **30**. Pearson correlation coefficients (*r*) are represented by a red–blue scale, with red indicating negative and blue indicating positive correlations (−1 to +1). Lipophilicity (log *P*) showed a strong negative correlation with 12Z viability (*r* = −0.73), whereas polarity-related descriptors (TPSA, HBD, and HBA) correlated positively (*r* = 0.77–0.83). In contrast, Ishikawa cell viability exhibited no significant association with the descriptors (*r* ≤ 0.26). Δviability (Ishikawa – 12Z) demonstrated weak but consistent correlations, being negatively associated with log *P* (*r* = −0.24) and positively associated with TPSA, HBD, and HBA (*r* = 0.19–0.25).

Finally, the molecular weight and ring count showed minimal variation (approximately 250–300 Da and two to three aromatic rings, respectively) and showed no meaningful correlation with either activity or selectivity. This indicates that within the studied chemical space, substituent-dependent parameters, including lipophilicity, polarity, hydrogen bonding, and electronic effects, are the primary determinants of fixed-dose selectivity outcomes. Overall, chalcone represents a tunable molecular scaffold in which subtle substituent modifications dictate whether selectivity follows a cytotoxic pathway (desired: reduced 12Z viability with preserved Ishikawa viability) or a cytoprotective pathway (undesired for therapy: high viability for both cell lines).

Across the series, compounds bearing nitro or chloro substituents (−NO_2_ and −Cl) and exhibiting higher lipophilicity were more frequently associated with reduced 12Z viability while maintaining a higher Ishikawa cell viability at the fixed dose. In contrast, compounds containing hydroxyl, methoxy, or amino substituents (−OH, −OMe, and −NR_2_), together with increased polarity-related descriptors such as TPSA and hydrogen-bonding capacity, tended to display lower cytotoxicity and, in some cases, cytoprotective or proliferative effects. This fixed-dose evaluation, based on the comparative response of endometriotic (12Z) and eutopic endometrial (Ishikawa) cells, provides a consistent operational definition of selectivity throughout the study and explains why compounds **24** and **28** emerge as the most selective candidates, with **14**, **17**, **23**, and **30** identified as additional statistically supported hits.

## 3 Experimental Section

### 3.1 General

Nuclear magnetic resonance (NMR) spectra were obtained using a Varian INOVA spectrometer, operating at 500 and 125 MHz for ^1^H and ^13^C nuclei, respectively, with CDCl_3_ or DMSO-*d*_6_ or CD_3_OD (Sigma-Aldrich) as the solvent and internal standards.

### 3.2 Source of Flavonoids **1**–**13**

Flavonoids **1**–**4**, **7**–**8**, and **10**–**11** were obtained commercially (Sigma-Aldrich, purity ≥95%) and used without further purification. Flavonoids **5**, **9**, and **12** were isolated from the DCM phase of MeOH extract from the leaves of *Baccharis lateralis*, while **6** and **13** were isolated from MeOH extract from the leaves of *Baccharis sphenophylla*, as previously reported.^43,44^ Compound identities were confirmed by comparison of their NMR data with reported literature values.

### 3.3 Synthesis of Chalcones **14**–**37**

Chalcones **14**–**37** were prepared by Claisen–Schmidt condensation as previously described.^29,31^ Compound identities were confirmed by comparison of their NMR data with reported literature values.

### 3.4 Cell Culture

Ishikawa and 12Z cells (Sigma-Aldrich) were cultured in 75 cm^2^ flasks at 37 °C and 5% CO_2_. Ishikawa cells used MEM with 5% FBS, and 12Z cells used high-glucose DMEM with 10% FBS. Both media were supplemented with penicillin (100 U/mL), streptomycin (10 U/mL), nonessential amino acids, and sodium pyruvate.

### 3.5 Cell Viability Assay

The MTT assay was employed to determine the cell viability. Ishikawa and 12Z cells were plated in 96-well plates at 4 × 10^4^ cells/cm^2^ and incubated for 24 h. Subsequently, cells were treated with compounds **1**–**37** at a concentration of 200 μM for 24 h. Stock solutions of all compounds were prepared in DMSO and diluted in culture medium to the desired concentration, resulting in a final DMSO concentration of 1% (v/v) in all treatments; control wells contained the same DMSO concentration. After that, 30 μL of MTT solution (5 mg/mL) was added to each well, and the plates were kept in the dark at 37 °C for 2 h. Results were expressed as cell viability % in comparison to untreated cells. All experiments were conducted in triplicate as independent assays. Analysis of variance (ANOVA) with Bonferroni’s test was used to evaluate the differences between cell groups (negative control versus treatment) for MTT experiments with a level of significance set at *p* < 0.05.

### 3.6 Molecular Descriptors and Computational Analysis

The chemical structures of compounds **1**–**37** were represented using SMILES notation and processed with the RDKit cheminformatics package (version 2024.09.6).^45^ From these structures, key physicochemical descriptors were calculated, including lipophilicity (log *P*, estimated by Crippen’s atom-additive MolLog P model and reported as dimensionless), topological polar surface area (TPSA, in Å^2^), number of hydrogen-bond donors (HBD) and acceptors (HBA), molecular weight (MW, Da), and total ring count (aromatic and aliphatic).^46,47^ All descriptor values were exported and are compiled in the Supporting Information, Table S1 to support structure–activity relationship (SAR) analysis.

The selectivity index was calculated as the difference in cell viability between Ishikawa and 12Z cells (Δviability = Ishikawa – 12Z), with positive values indicating selective toxicity toward 12Z cells. Correlations between physicochemical descriptors and biological activity were evaluated using Pearson’s correlation coefficient (*r*), and the corresponding coefficients of determination (*R* ^2^) were computed using GraphPad Prism 10 and were represented as a heatmap.^48,49^

Hierarchical clustering was performed in Python (scipy.cluster.hierarchy, v1.14.1) using Ward’s linkage and Euclidean distance to examine similarity relationships among the compounds based on the selected physicochemical descriptors.^50^ The resulting dendrogram was used to define six clusters, which were subsequently employed as a descriptive framework to organize and discuss the observed biological response patterns.^46^ Structural features, including common substituents, are described within each cluster solely to aid qualitative interpretation without being used as criteria for cluster definition or boundary assignment.

## 4 Conclusions

In this study, we successfully evaluated a library of 37 flavonoid derivatives, primarily chalcones, for their potential as selective therapeutic agents against endometriosis. By employing a comparative screening approach using 12Z endometriotic cells and Ishikawa normal endometrial cells, differential biological response patterns were observed across the compound series. These findings indicate that variations in physicochemical properties are associated with shifts between cytotoxic and cytoprotective phenotypes without supporting the establishment of a distinct structure–activity relationship. Derivatives featuring multiple hydroxyl or methoxy groups, high polarity (TPSA > 80 Å^2^), and extensive hydrogen-bonding capacity consistently exhibited cytoprotective effects, particularly in normal endometrial cells. Conversely, several chalcones bearing nitro or chloro substituents and exhibiting higher lipophilicity were among the compounds that showed increased cytotoxicity at a fixed dose.

Among the evaluated compounds, compounds **24** and **28** emerged as the most selective within the series. At the fixed dose, both compounds reduced the viability of endometriotic 12Z cells to approximately 50%, while preserving or, in some cases, increasing the viability of Ishikawa cells. This differential response characterizes a cytotoxic selectivity profile, in which activity is preferentially directed toward endometriotic cells rather than reflecting nonspecific cytotoxicity. Such a profile is particularly relevant in the context of endometriosis, where selective targeting of ectopic endometrial tissues remains a major therapeutic challenge.

Collectively, this work highlights chalcones as versatile scaffolds for the exploration of bioactive compounds with selective effects in endometriosis-related cellular models. The insights gained from the correlation between physicochemical descriptors (log *P* and TPSA) and biological outcomes provide a useful framework to guide future exploratory studies aimed at identifying more potent and selective compounds, paving the way for novel, nonhormonal therapeutic strategies for endometriosis management.

## Supplementary Material



## Notes

Table S1 reporting physicochemical descriptors and cell viability data for compounds **1**–**37**; NMR spectra (^1^H and ^13^C) of selected compounds (PDF)
