# Supplementary material for: Activity–Selectivity of Flavonoid Derivatives in Endometriotic Cells
Source: ACS Omega. 2026 Feb 2;11(6):10703–11. doi: 10.1021/acsomega.5c12546 (PMC12917715; doi:10.1021/acsomega.5c12546)
Supplement: Supplementary file 1 [file ao5c12546_si_002.pdf]

Activity-selectivity of flavonoid derivatives in endometriotic cells

Kaio S Gomes[1], Julia A Coelho[1], Pedro E H Tesser[2], Dalete C S Souza[2], Matheus L Silva[2], Edgar A Ferreira[3], Joao H G Lago[2], Giselle Cerchiaro[1*]

[1]Metal Biochemistry and Oxidative Stress Laboratory, Centre for Natural Sciences and Humanities, Federal University of ABC, Santo Andre – SP, Brazil. 09210-5800

[2]Laboratory of Chemical Biology, Centre for Natural Sciences and Humanities, Federal University of ABC, Santo Andre – SP, Brazil. 09210-580

[3]School of Engineering, Mackenzie Presbyterian University, São Paulo – SP, Brazil. 01302-907

*corresponding author (Tel +55 11 49960160, E-mail giselle.cerchiaro@ufabc.edu.br)

Content Table

Table S **1** - Physicochemical descriptors and cell viability data for compounds 1–37. SMILES-encoded structures were processed with RDKit to calculate Log P, TPSA, HBD, HBA, MW, and ring count. Cell viability in Ishikawa and 12Z cells (%, ±SD) and the corresponding selectivity index (Δ viability) are reported to support structure–activity relationship (SAR) analysis......4

Figure S 1 - ^{1}H NMR Spectrum for Compound **5** (δ, DMSO-d_6, 500 MHz)......6
Figure S 2 - ^{13}C Spectrum for Compound 5 (δ, DMSO-d_6, 125 MHz)......7
Figure S 3 - ^{1}H Spectrum for Compound **6** (δ, CD_3OD, 500 MHz)......8
Figure S 4 - ^{13}C Spectrum for Compound **6** (δ, CD_3OD, 125 MHz)......9
Figure S 5 - ^{1}H NMR Spectrum for Compound **9** (δ, DMSO-d_6, 500 MHz)......10
Figure S 6 - ^{13}C NMR Spectrum for Compound **9** (δ, DMSO-d_6, 125 MHz)......11
Figure S 7 - ^{1}H NMR Spectrum for Compound **12** (δ, DMSO-d_6, 125 MHz)......12
Figure S 8 - ^{13}C NMR Spectrum for Compound **12** (δ, DMSO-d_6, 125 MHz)......13
Figure S 9 - ^{1}H NMR Spectrum for Compound **13** (δ, CD_3OD, 500 MHz)......14
Figure S 10 - ^{13}C NMR Spectrum for Compound **13** (δ, CD_3OD, 125 MHz)......15
Figure S 11 - ^{1}H NMR Spectrum of Compound **14** (δ, $CDCl_3$, 500 MHz)......16
Figure S 12 - ^{1}H NMR Spectra of Compound **15** (δ, $CDCl_3$, 500 MHz)......17
Figure S 13 - ^{1}H NMR Spectra of Compound **16** (δ, $CDCl_3$, 500 MHz)......18
Figure S 14 - ^{1}H NMR Spectra of Compound **17** (δ, $CDCl_3$, 500 MHz)......19
Figure S 15 - ^{1}H NMR Spectrum of Compound **18** (δ, $CDCl_3$, 500 MHz)......20
Figure S 16 - ^{1}H NMR Spectra of Compound **19** (δ, $CDCl_3$, 500 MHz)......21
Figure S 17 - ^{1}H NMR Spectra of Compound **20** (δ, $CDCl_3$, 500 MHz)......22
Figure S 18 - ^{1}H NMR Spectrum of Compound **21** (δ, $CDCl_3$, 500 MHz)......23
Figure S 19 - ^{1}H NMR Spectrum of Compound **22** (δ, $CDCl_3$, 500 MHz)......24
Figure S 20 - ^{1}H NMR Spectrum of Compound **23** (δ, $CDCl_3$, 500 MHz)......25
Figure S 21 - ^{1}H NMR Spectrum of Compound **24** (δ, $CDCl_3$, 500 MHz)......26
Figure S 22 - ^{1}H NMR Spectrum of Compound **25** (δ, $CDCl_3$, 500 MHz)......27

Figure S 23 - ^{1}H NMR Spectrum of Compound **26** (δ, $CDCl_3$, 500 MHz).............28
Figure S 24 - ^{1}H NMR Spectrum of Compound **27** (δ, $CDCl_3$, 500 MHz).............29
Figure S 25 - ^{1}H NMR Spectrum of Compound **28** (δ, $CDCl_3$, 500 MHz).............30
Figure S 26 - ^{1}H NMR Spectrum of Compound **29** (δ, $CDCl_3$, 500 MHz).............31
Figure S 27 - ^{1}H NMR Spectrum of Compound **30** (δ, $CDCl_3$, 500 MHz).............32
Figure S 28 - ^{1}H NMR Spectrum of Compound **31** (δ, $CDCl_3$, 500 MHz).............33
Figure S 29 - ^{1}H NMR Spectrum of Compound **32** (δ, $CDCl_3$, 500 MHz).............34
Figure S 30 - ^{1}H NMR Spectrum of Compound **33** (δ, $CDCl_3$, 500 MHz).............35
Figure S 31 - ^{1}H NMR Spectrum of Compound **34** (δ, $CDCl_3$, 500 MHz).............36
Figure S 32 - ^{1}H NMR Spectrum of Compound **35** (δ, $CDCl_3$, 500 MHz).............37
Figure S 33 - ^{1}H NMR Spectrum of Compound **36** (δ, $CDCl_3$, 500 MHz).............38
Figure S 34 - ^{1}H NMR Spectrum of Compound **37** (δ, $CDCl_3$, 500 MHz).............39

Table S **1** - Physicochemical descriptors and cell viability data for compounds 1–37. SMILES-encoded structures were processed with RDKit to calculate Log P, TPSA, HBD, HBA, MW, and ring count. Cell viability in Ishikawa and 12Z cells (%, ±SD) and the corresponding selectivity index (Δ viability) are reported to support structure–activity relationship (SAR) analysis

Compound	SMILES	Cell Viability (% ± SD) Ishikawa	Cell Viability (% ± SD) 12Z	Δ Viability	Log P	TPSA (Å²)	HBD	HBA	MW (Da)	Ring Count
**1**	O=C1C=C(C2=CC=C(OC)C(OC)=C2)OC3=C1C(O)=CC(OC)=C3	155.36 ± 29.73	91.19 ± 1.20	64.17	3.19	78.13	1	6		3
**2**	O=C1C=C(C2=CC=C(O)C(O)=C2)OC3=C1C(O)=CC(O)=C3	150.36 ± 12.81	109.02 ± 11.12	41.34	2.28	111.13	4	6	286.24	3
**3**	O=C1C=C(C2=CC=CC=C2)OC3=C1C(O)=CC=C3	64.94 ± 5.17	ND	ND	3.17	50.44	1	3	238.24	3
**4**	O=C1CC(C2=CC=C(O)C=C2)OC3=C1C(O)=CC(O)=C3	63.25 ± 9.98	ND	ND	2.51	86.99	3	5	272.26	3
**5**	O=C1CC(C2=CC=C(O)C=C2)OC3=C1C(O)=CC(OC)=C3	83.38 ± 7.03	81.96 ± 4.56	1.42	2.81	75.99	2	5	286.28	3
**6**	O=C1C=C(C2=CC=C(O)C(O)=C2)OC3=C1C=C(OC)C(O)=C3	101.97 ± 14.33	85.98 ± 4.48	15.99	2.59	100.13	3	6	300.27	3
**7**	OC1=C(C2=CC=C(O)C(O)=C2)OC3=C(C(O)=CC(O)=C3)C1=O	149.41 ± 10.81	128.41 ± 0.06	21.00	1.99	131.36	5	7	302.24	3
**8**	O=C1C=C(C2=CC=CC=C2)OC3=C1C=CC(O)=C3	126.80 ± 2.26	88.53 ± 15.41	38.27	3.17	50.44	1	3	238.24	3
**9**	O=C1C=C(C2=CC=C(O)C=C2)OC3=C1C(O)=CC(O)=C3	58.52 ± 13.36	ND	ND	2.58	90.90	3	5	270.24	3
**10**	O=C1C(O)=C(C2=CC=CC=C2)OC3=C1C=CC(O)=C3	52.78 ± 1.85	ND	ND	2.87	70.67	2	4	254.24	3
**11**	O=C1C(O)=C(C2=CC=CC=C2)OC3=C1C=CC=C3	32.19 ± 5.81	ND	ND	3.17	50.44	1	3	238.24	3
**12**	OC1=C(C2=CC(O)=C(O)C(O)=C2)OC3=C(C(O)=CC(O)=C3)C1=O	73.70 ± 2.30	122.73 ± 3.30	-49.03	1.69	151.59	6	8	318.24	3
**13**	O=C1C=C(C2=CC=C(O)C=C2)OC3=C1C(O)=C(OC)C(O)=C3	87.28 ± 4.36	78.11 ± 14.28	9.17	2.59	100.13	3	6	300.27	3
**14**	O=C(C1=CC=CC=C1)/C=C/C2=CC(OCO3)=C3C=C2	81.93 ± 3.10	59.90 ± 4.11	22.03	3.31	35.53	0	3	252.27	3
**15**	O=C(C1=CC=CC=C1O)/C=C/C2=CC(OCO3)=C3C=C2	78.11 ± 3.09	50.64 ± 7.26	27.47	3.02	55.76	1	4	268.27	3

**16**	O=C(C1=CC=C(OC)C=C1)/C=C/C2=CC(OCO3)=C3C=C2	55.87 ± 4.54	ND	ND	3.32	44.76	0	4	282.30	3
**17**	O=C(C1=CC=C([N+]([O-])=O)C=C1)/C=C/C2=CC(OCO3)=C3C=C2	127.05 ± 3.05	73.63 ± 6.06	53.42	3.22	78.67	0	5	297.27	3
**18**	O=C(C1=CC=CC=C1)/C=C/C2=CC=CC=C2	9.09 ± 2.24	ND	ND	3.58	17.07	0	1	208.26	2
**19**	O=C(C1=CC=CC=C1O)/C=C/C2=CC=CC=C2	40.52 ± 6.55	ND	ND	3.29	37.30	1	2	224.26	2
**20**	O=C(C1=CC=C(OC)C=C1)/C=C/C2=CC=CC=C2	10.75 ± 1.43	ND	ND	3.59	26.30	0	2	238.29	2
**21**	O=C(C1=CC=CC=C1)/C=C/C2=CC=C(N(C)C)C=C2	47.85 ± 2.14	ND	ND	3.65	20.31	0	2	251.33	2
**22**	O=C(C1=CC=C([N+]([O-])=O)C=C1)/C=C/C2=CC=C(N(C)C)C=C2	64.21 ± 7.84	ND	ND	3.56	63.45	0	4	296.33	2
**23**	O=C(C1=CC=CC=C1O)/C=C/C2=CC=C(N(C)C)C=C2	84.51 ± 10.28	58.93 ± 5.40	25.58	3.35	40.54	1	3	267.33	2
**24**	O=C(C1=CC=CC(O)=C1)/C=C/C2=CC=C(N(C)C)C=C2	109.23 ± 33.65	48.65 ± 1.29	60.58	3.35	40.54	1	3	267.33	2
**25**	O=C(C1=CC=CC(N)=C1)/C=C/C2=CC=C(N(C)C)C=C2	64.08 ± 12.98	ND	ND	3.23	46.33	1	3	266.34	2
**26**	O=C(C1=CC=C(Cl)C=C1)/C=C/C2=CC=C(N(C)C)C=C2	80.82 ± 12.51	79.80 ± 10.70	1.02	4.30	20.31	0	2	285.77	2
**27**	O=C(C1=CC=C(OC)C=C1)/C=C/C2=CC=C(N(C)C)C=C2	29.95 ± 5.36	ND	ND	3.66	29.54	0	3	281.36	2
**28**	O=C(C1=CC=C(Cl)C=C1)/C=C/C2=CC(OCO3)=C3C=C2	114.67 ± 3.33	53.18 ± 5.51	61.49	3.96	35.53	0	3	286.71	3
**29**	O=C(C1=CC=CC(O)=C1)/C=C/C2=CC(OCO3)=C3C=C2	12.48 ± 1.43	ND	ND	3.02	55.76	1	4	268.27	3
**30**	O=C(C1=CC=CC=C1)/C=C/C2=CC(OC)=C(OC)C(OC)=C2	114.07 ± 10.87	69.91 ± 5.40	44.16	3.61	44.76	0	4	298.34	2
**31**	O=C(C1=CC=C(C2=CC=CC=C2)C=C1)/C=C/C3=CC(OC)=C(OC)C(OC)=C3	23.33 ± 4.68	ND	ND	5.28	44.76	0	4	374.44	3
**32**	O=C(C1=CC=C(C2=CC=CC=C2)C=C1)/C=C/C3=CC=CC=C3	46.08 ± 10.69	ND	ND	5.25	17.07	0	1	284.36	3
**33**	O=C(C1=CC=C(OC)C=C1)/C=C/C2=CC=C(Cl)C=C2	45.73 ± 12.78	ND	ND	4.24	26.30	0	2	272.73	2
**34**	O=C(C1=CC=C(OC)C=C1)/C=C/C2=CC=C([N+]([O-])=O)C=C2	43.58	ND	ND	3.50	69.44	0	4	283.28	2
**35**	O=C(C1=CC=CC=C1OCC=C)/C=C/C2=CC(OCO3)=C3C=C2	46.80 ±3.63	ND	ND	3.88	44.76	0	4	308.33	3
**36**	O=C(C1=CC=C([N+]([O-])=O)C=C1)/C=C/C2=CC=CC=C2	59.49 ± 8.40	ND	ND	3.49	60.21	0	3	253.26	2
**37**	O=C(C1=CC=CC(O)=C1)/C=C/C2=CC=CC=C2	57.01 ± 7.44	ND	ND	3.29	37.30	1	2	224.26	2

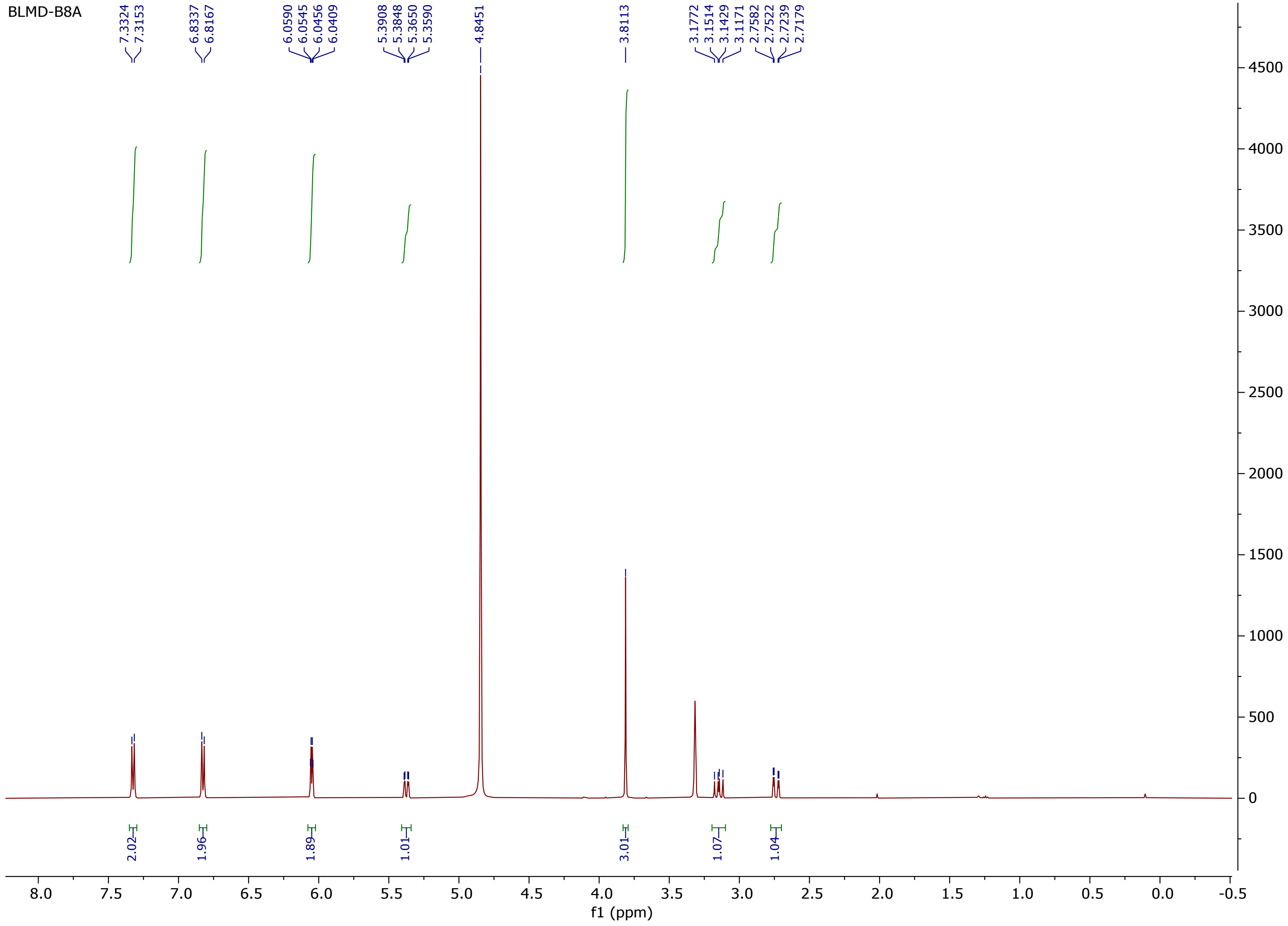

Figure S 1 - ^{1}H NMR Spectrum for Compound **5** (δ, DMSO-d_6, 500 MHz)

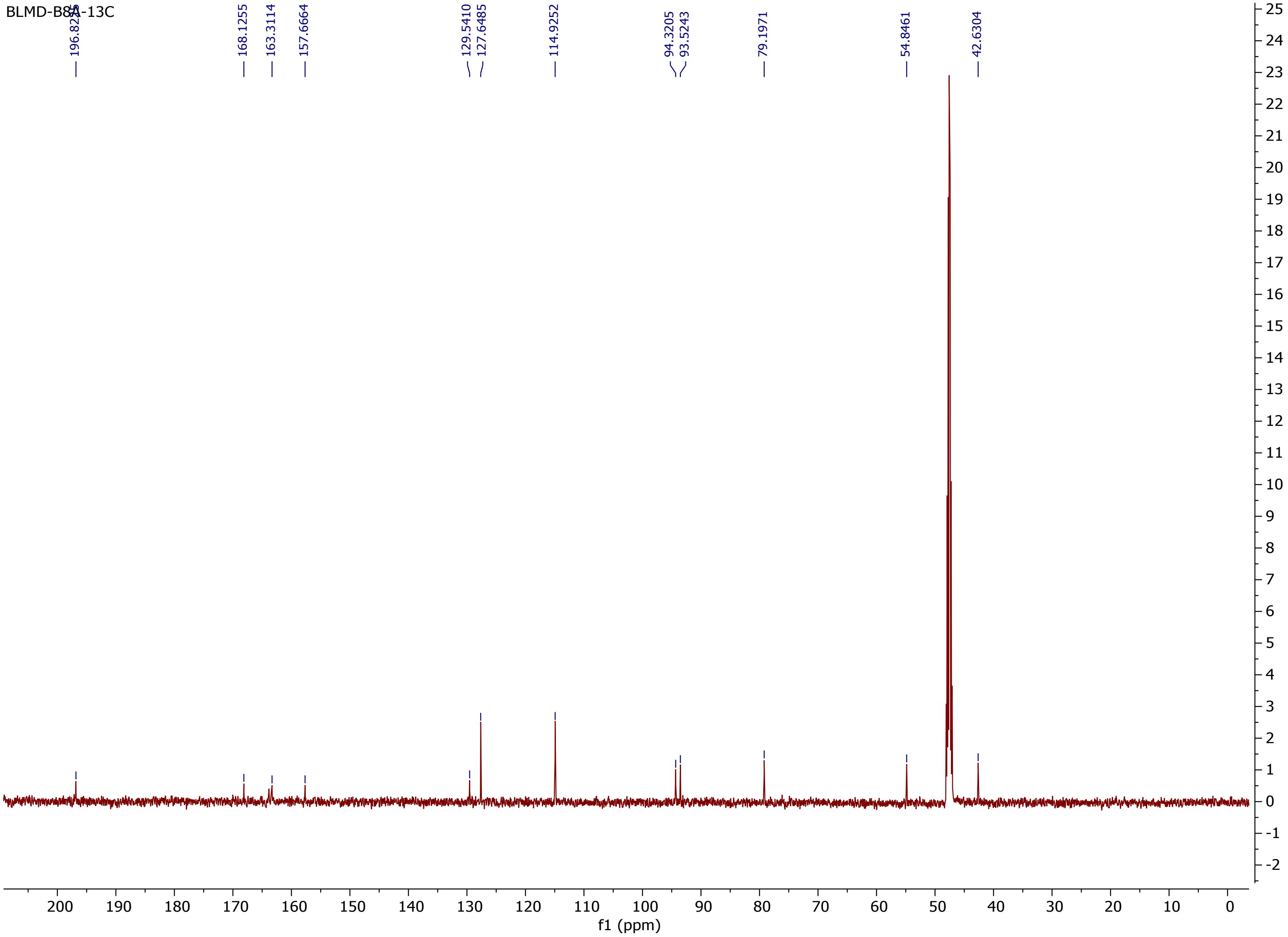

Figure S 2 - ^{13}C Spectrum for Compound 5 (δ, DMSO-d_6, 125 MHz)

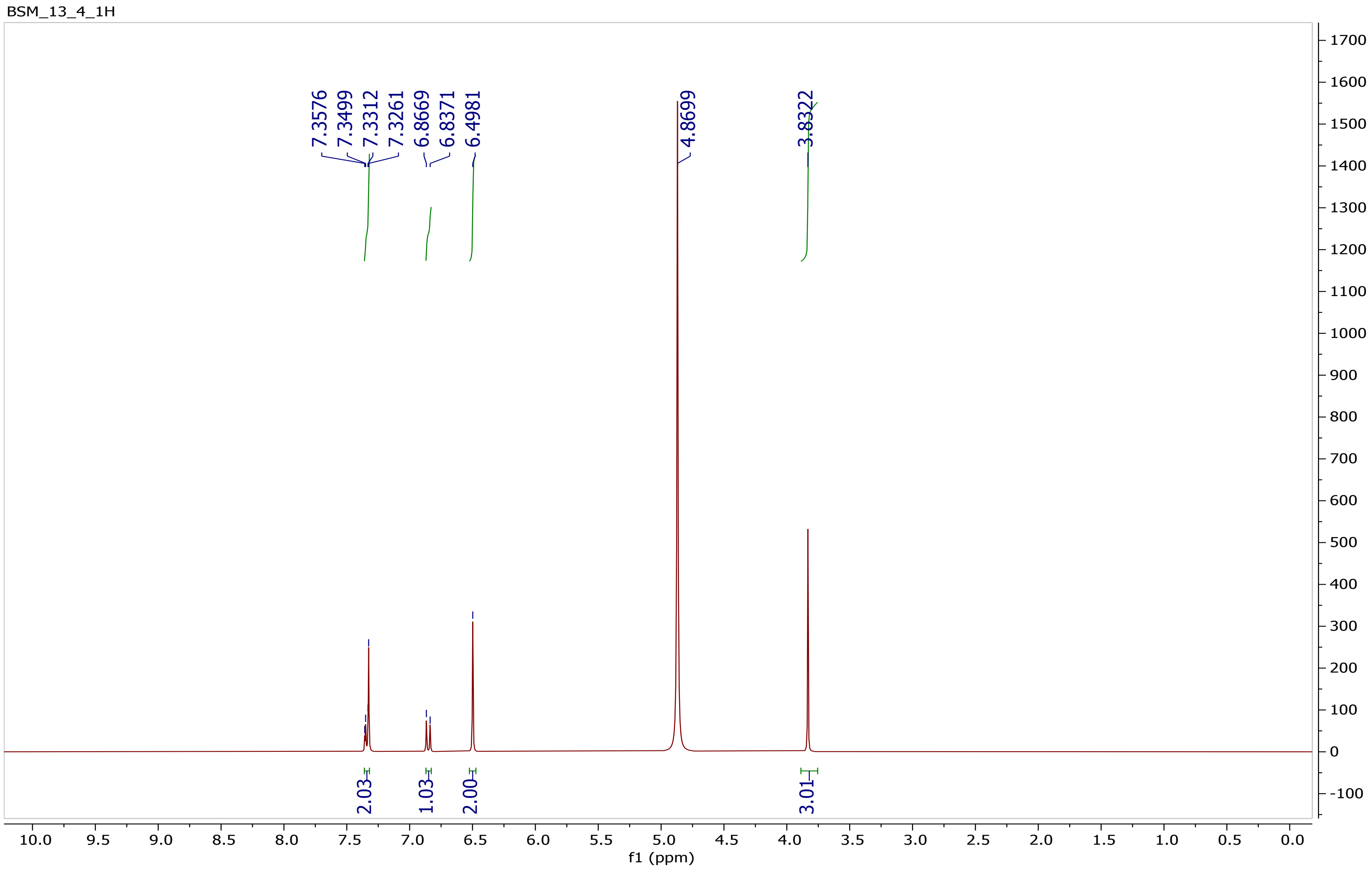

Figure S 3 - ^{1}H Spectrum for Compound **6** (δ, CD_3OD, 500 MHz)

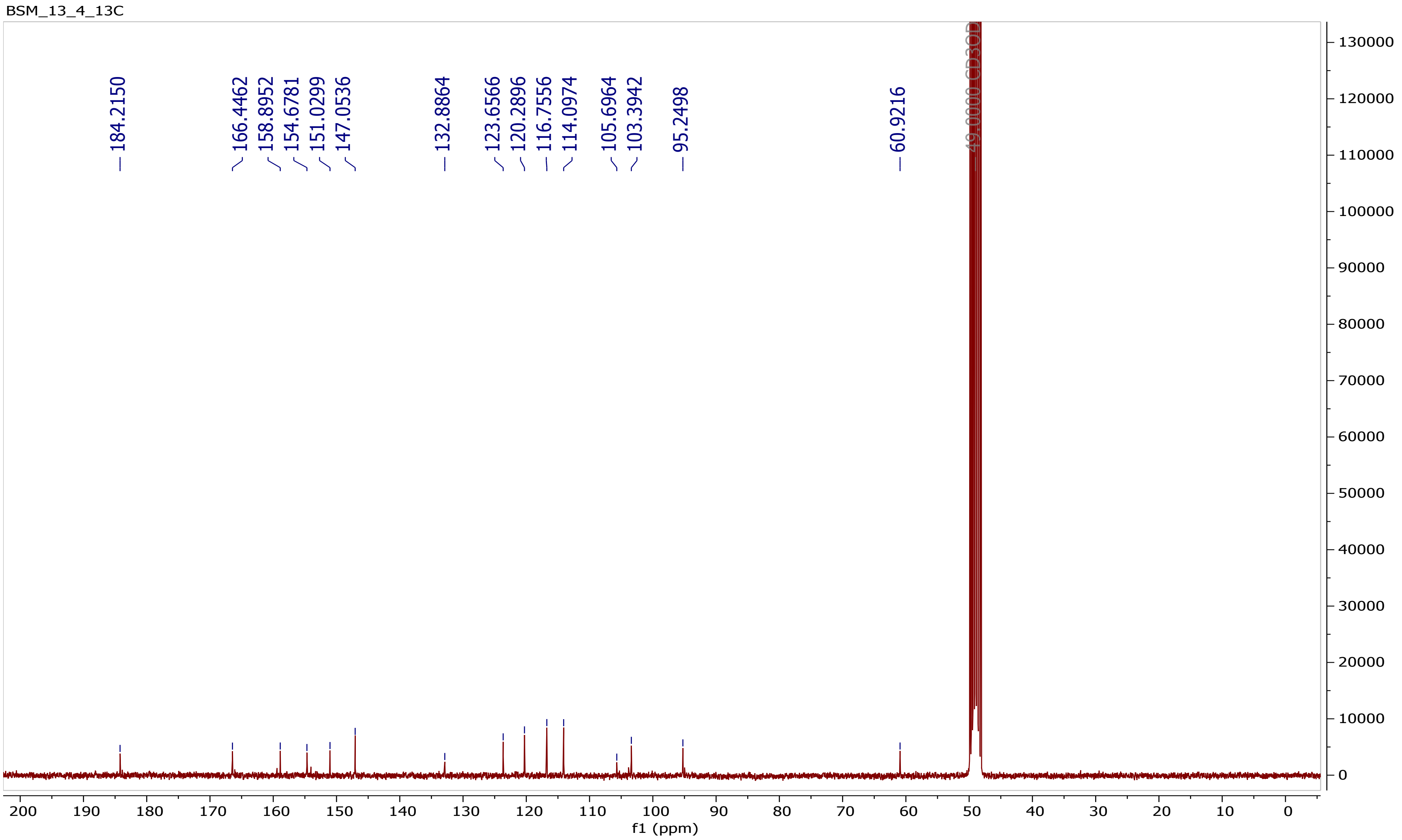

Figure S 4 - ^{13}C Spectrum for Compound **6** (δ, CD_3OD, 125 MHz)

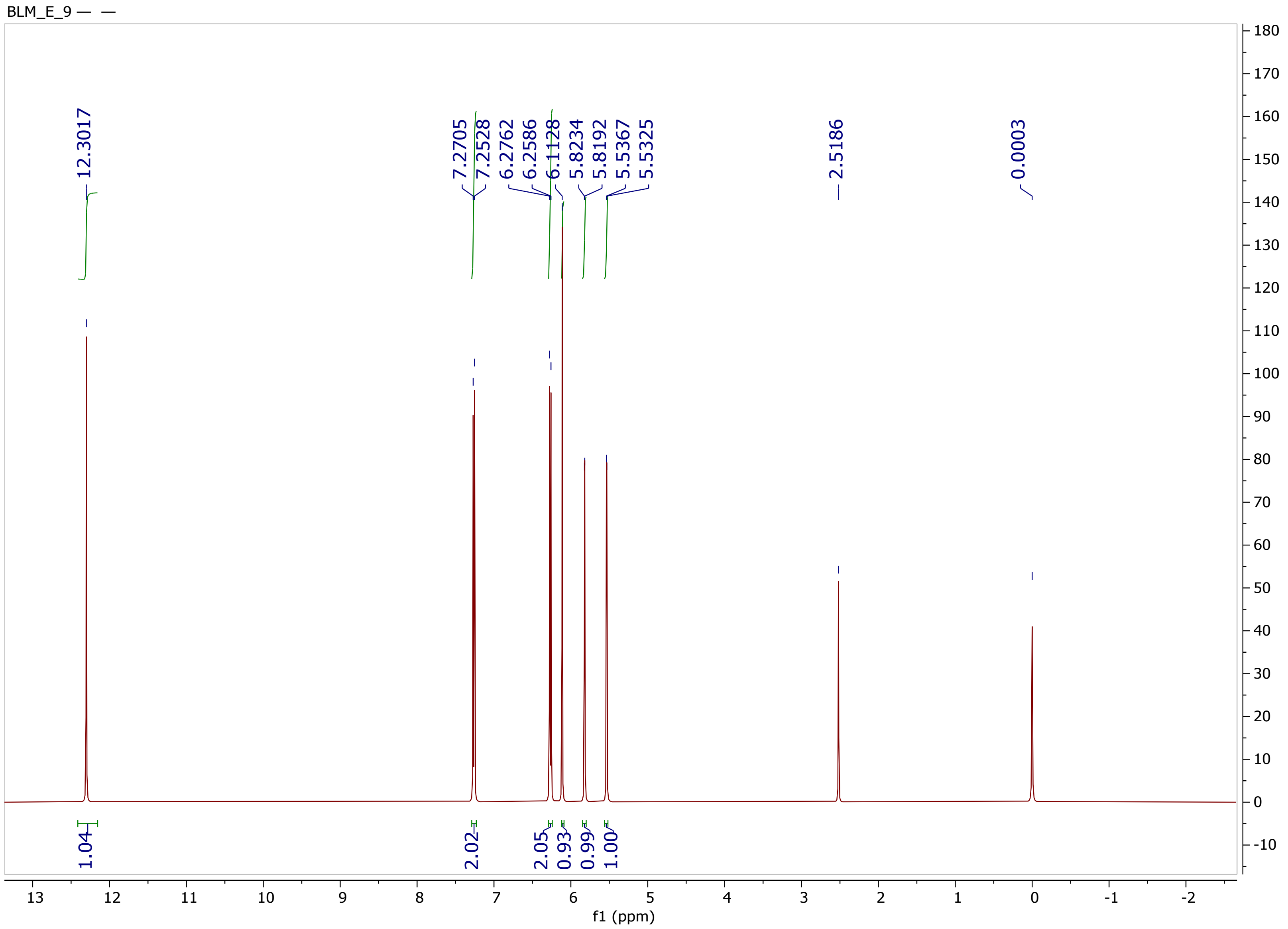

Figure S 5 - ^{1}H NMR Spectrum for Compound **9** (δ, DMSO-d_6, 500 MHz)

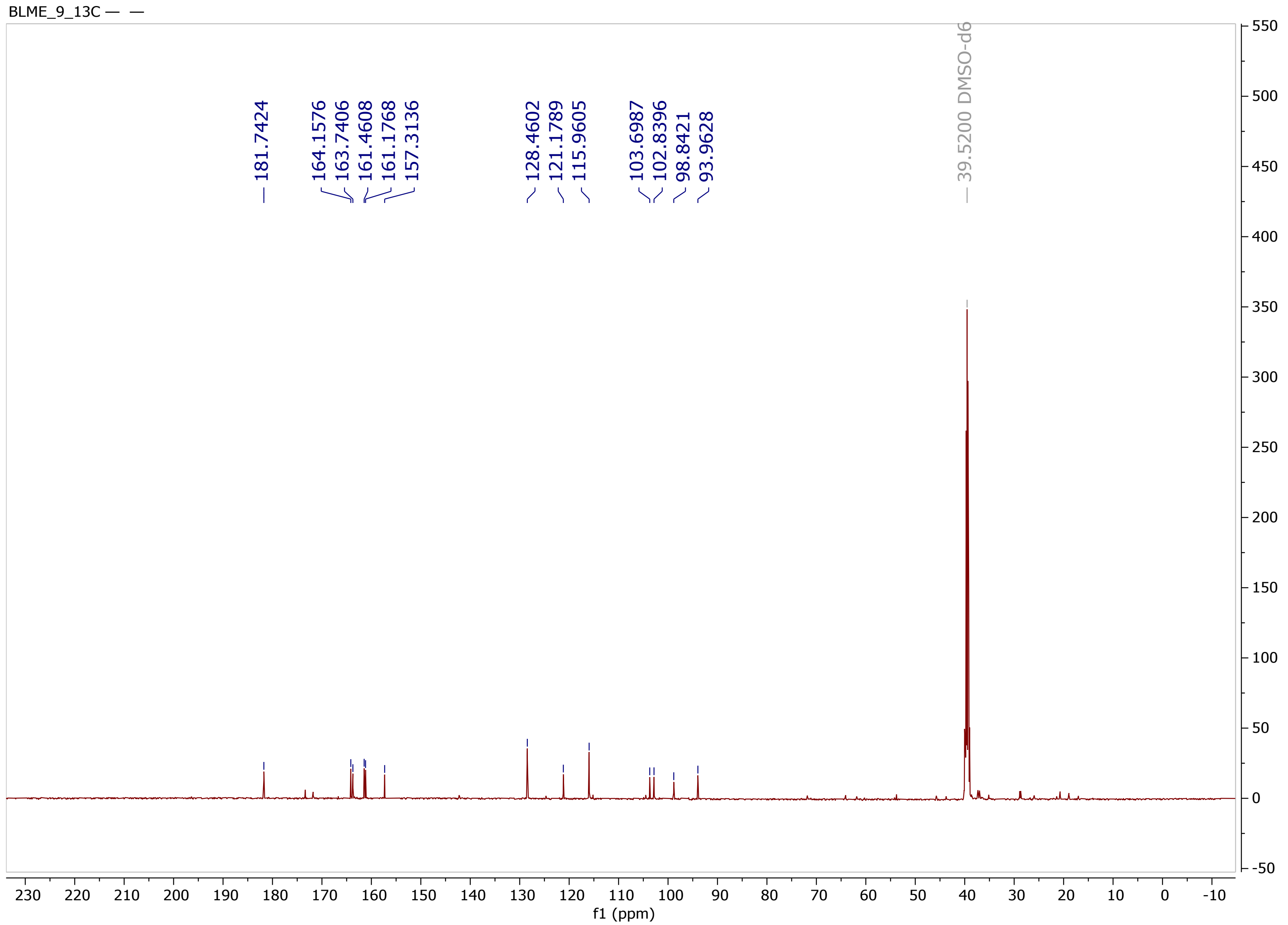

Figure S 6 - ^{13}C NMR Spectrum for Compound **9** (δ, DMSO-d_6, 125 MHz)

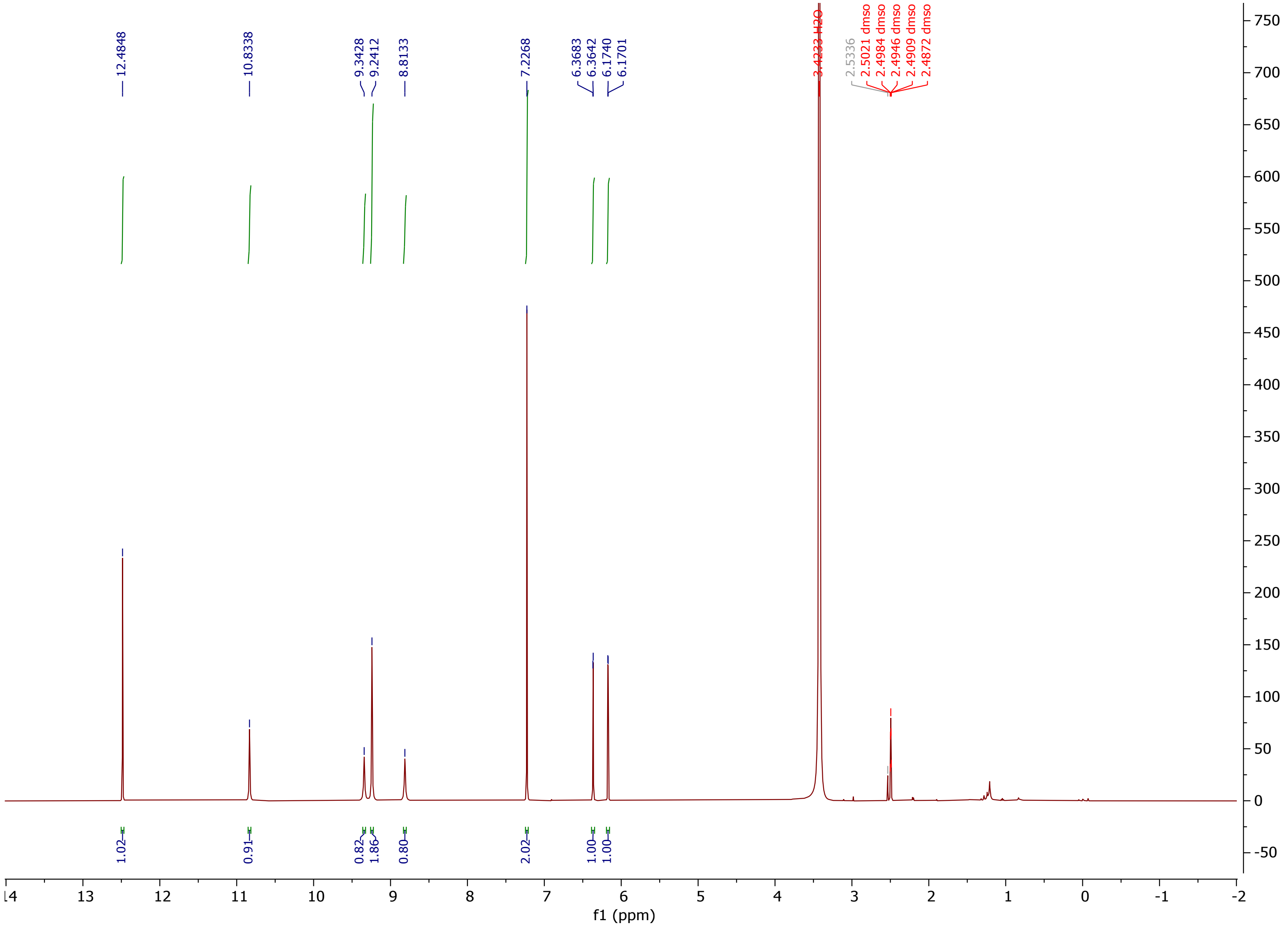

Figure S 7 - ^{1}H NMR Spectrum for Compound **12** (δ, DMSO-d_6, 125 MHz)

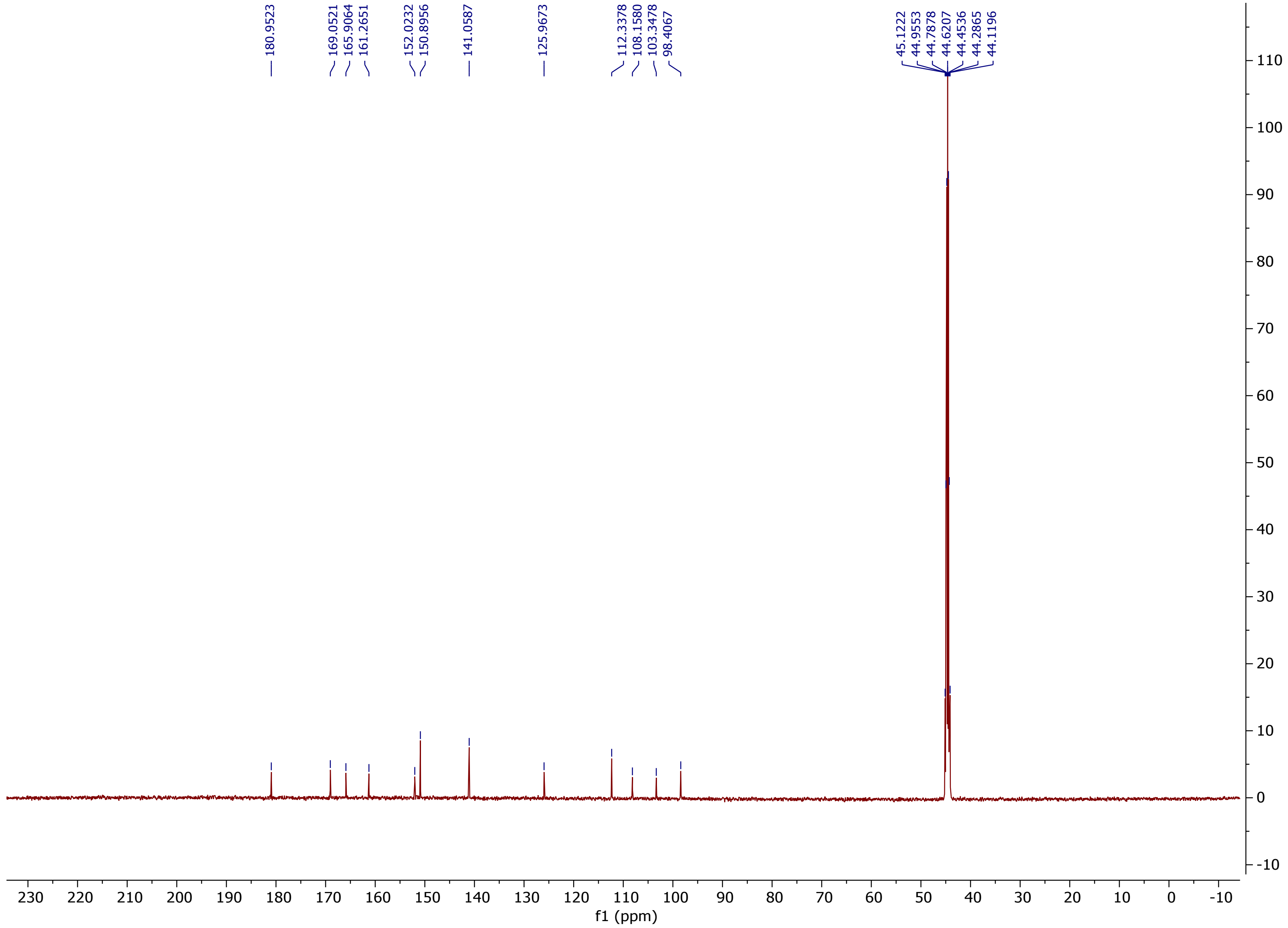

Figure S 8 - ^{13}C NMR Spectrum for Compound **12** (δ, DMSO-d_6, 125 MHz)

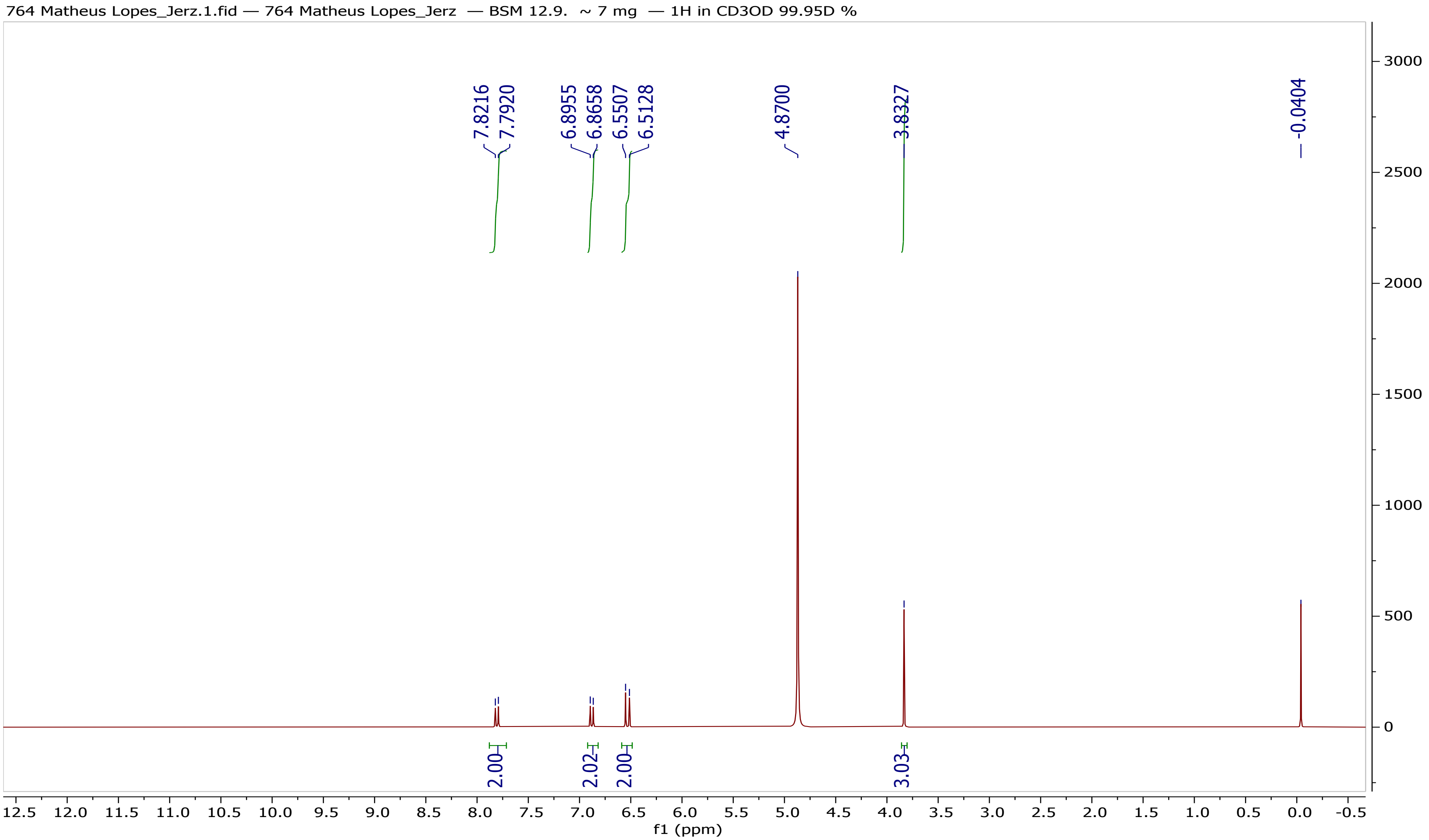

Figure S 9 - ^{1}H NMR Spectrum for Compound **13** (δ, CD_3OD, 500 MHz)

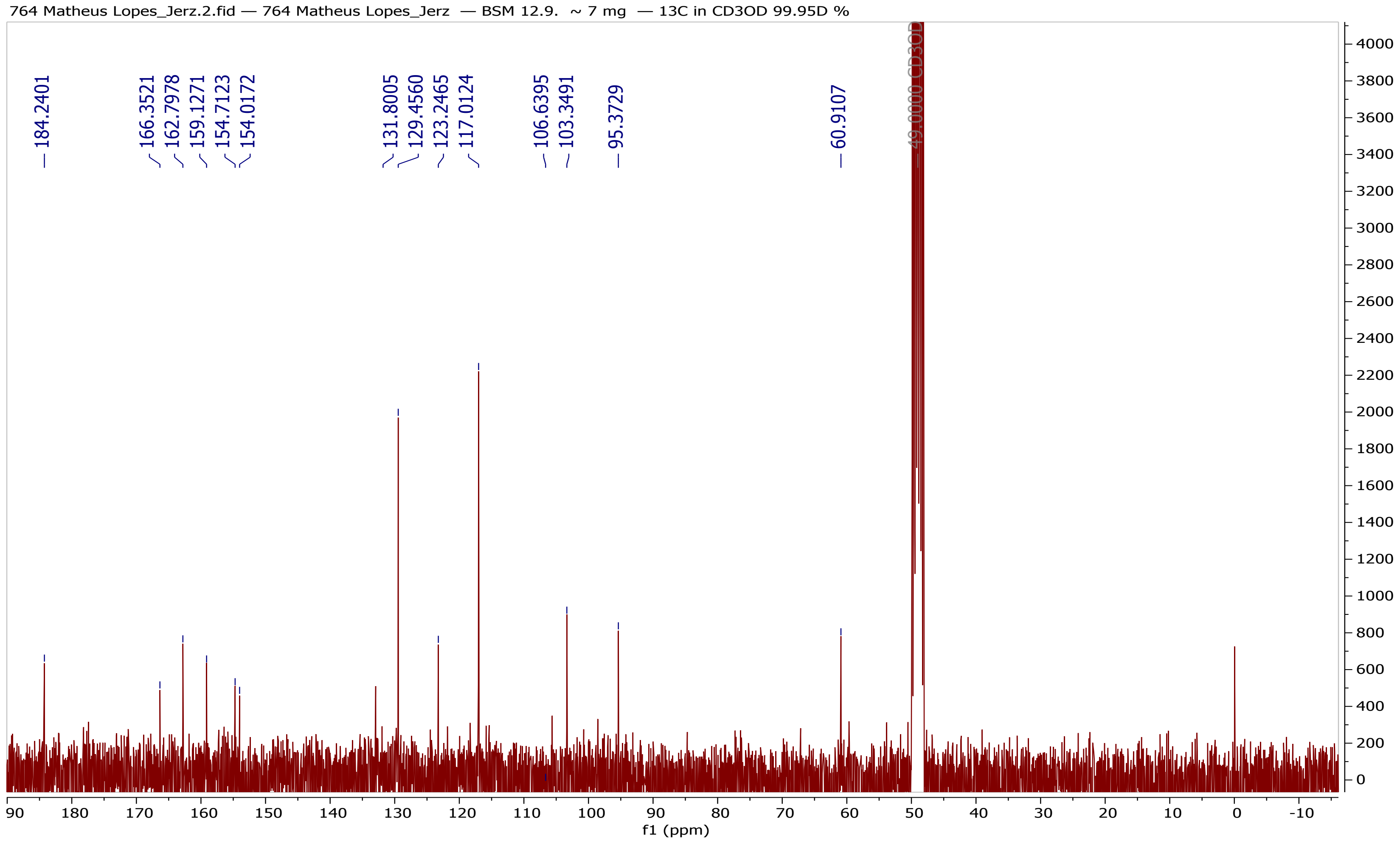

Figure S 10 - ^{13}C NMR Spectrum for Compound **13** (δ, CD_3OD, 125 MHz)

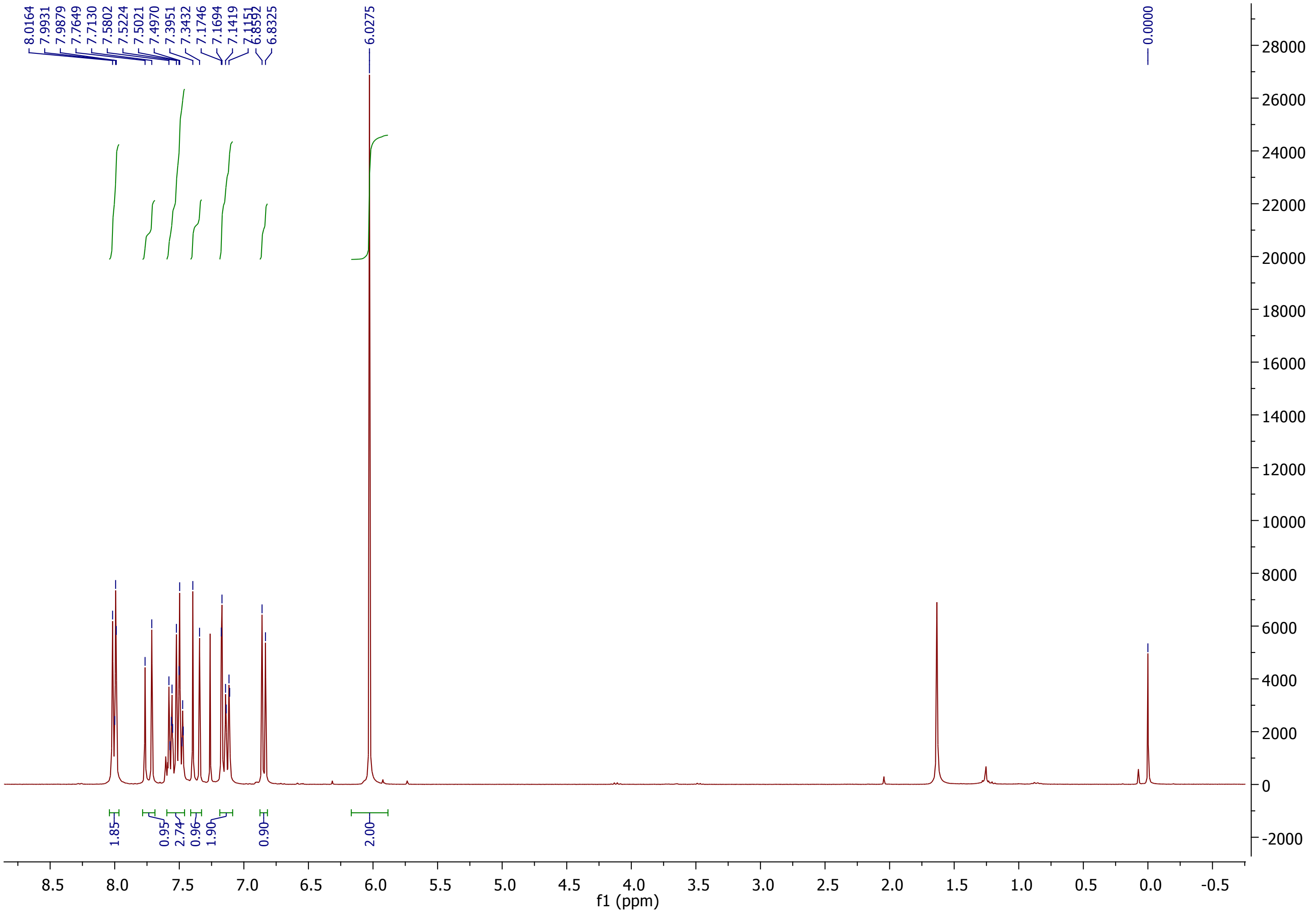

Figure S 11 - ^{1}H NMR Spectrum of Compound **14** (δ, $CDCl_3$, 500 MHz)

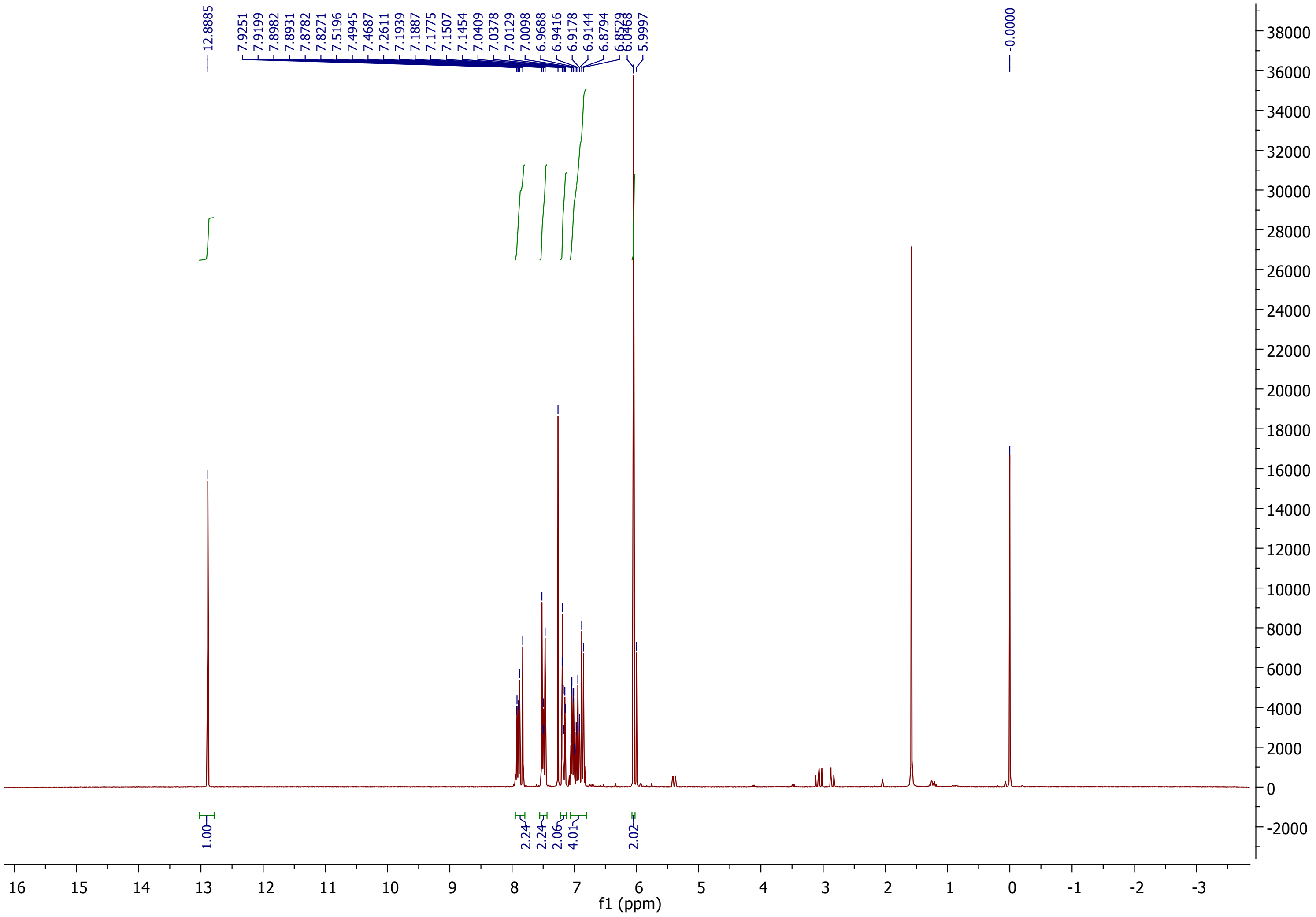

Figure S 12 - ^{1}H NMR Spectra of Compound **15** (δ, $CDCl_3$, 500 MHz)

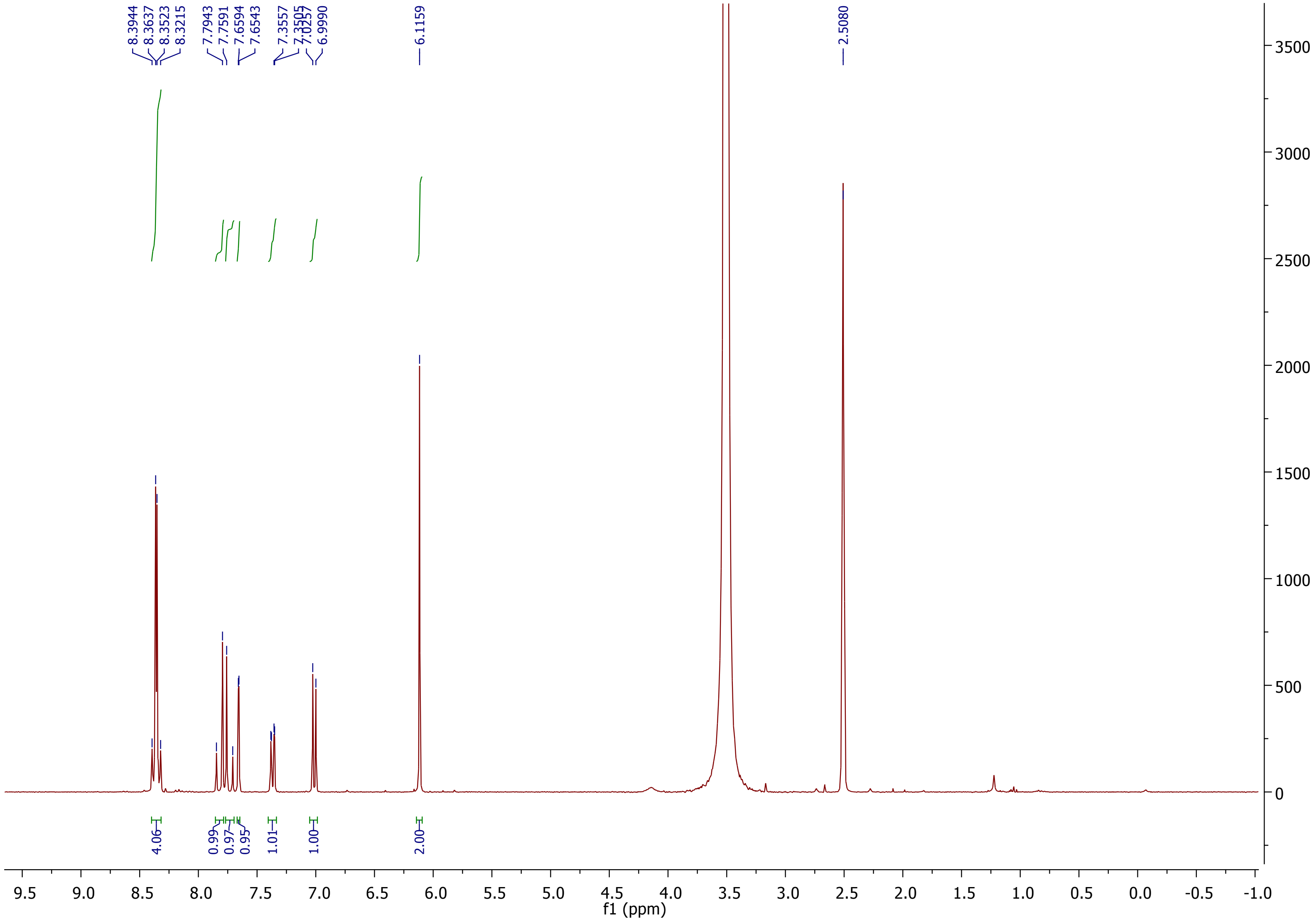

Figure S 13 - ^{1}H NMR Spectra of Compound **16** (δ, $CDCl_3$, 500 MHz)

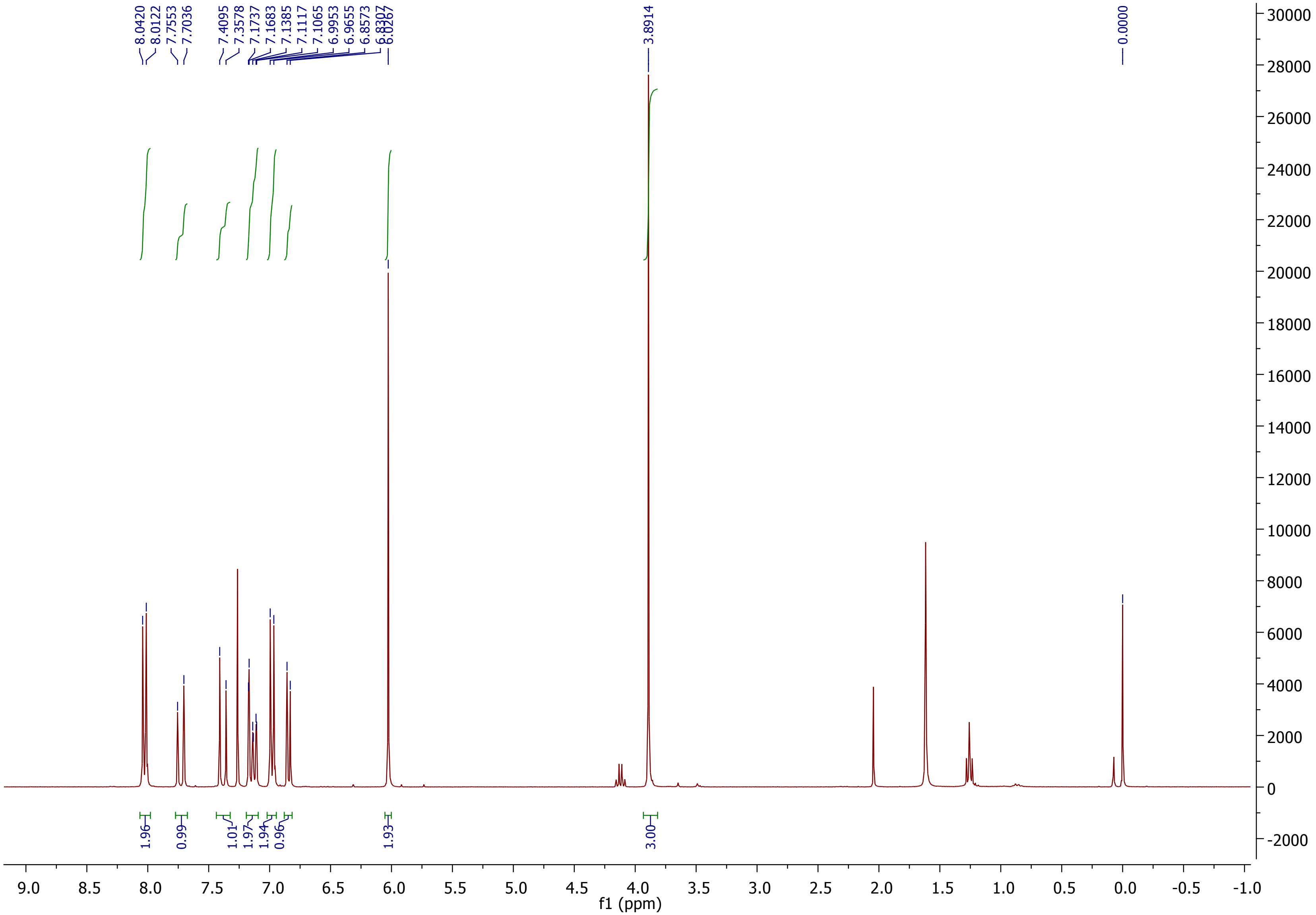

Figure S 14 - ^{1}H NMR Spectra of Compound **17** (δ, $CDCl_3$, 500 MHz)

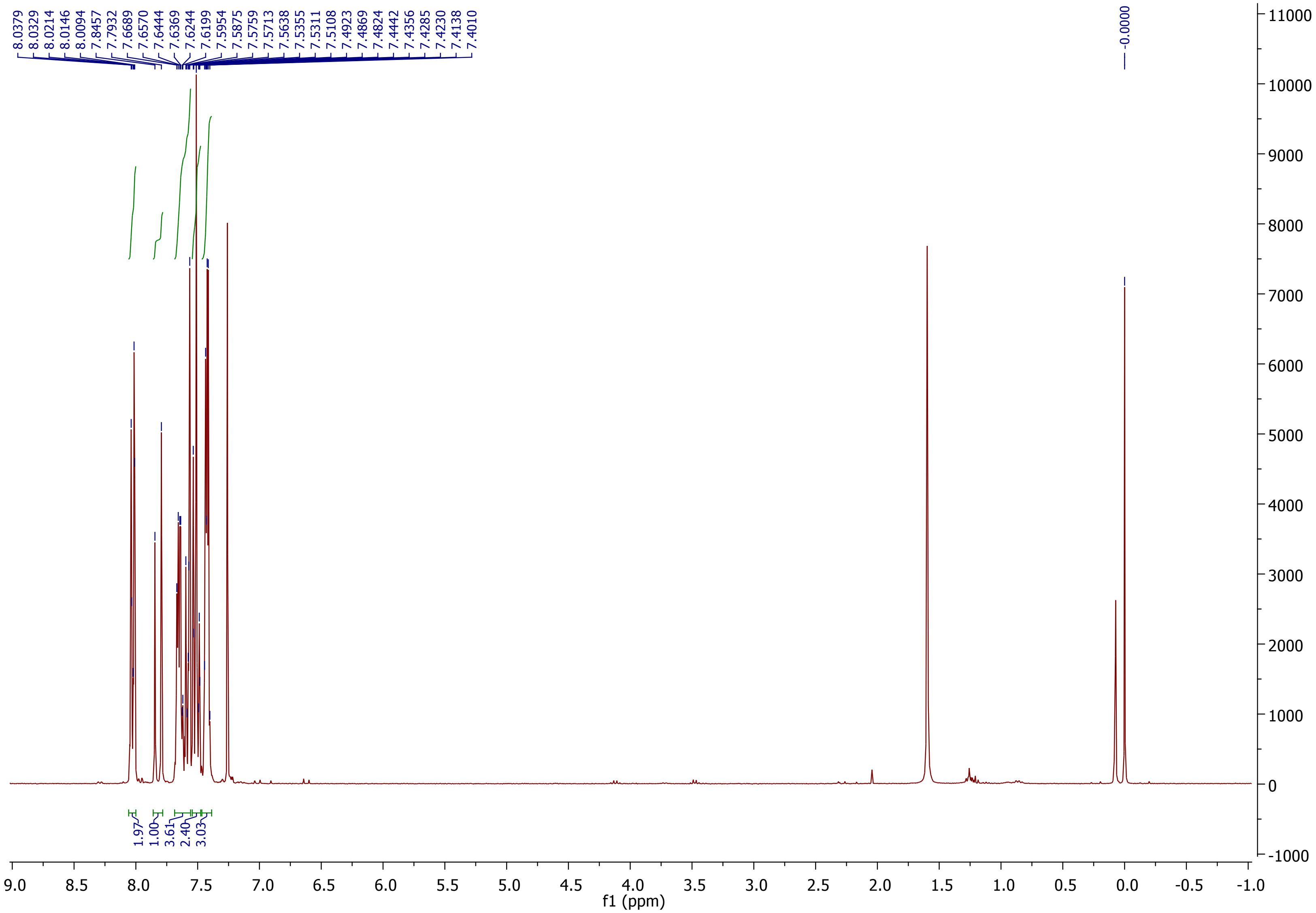

Figure S 15 - ^{1}H NMR Spectrum of Compound **18** (δ, $CDCl_3$, 500 MHz)

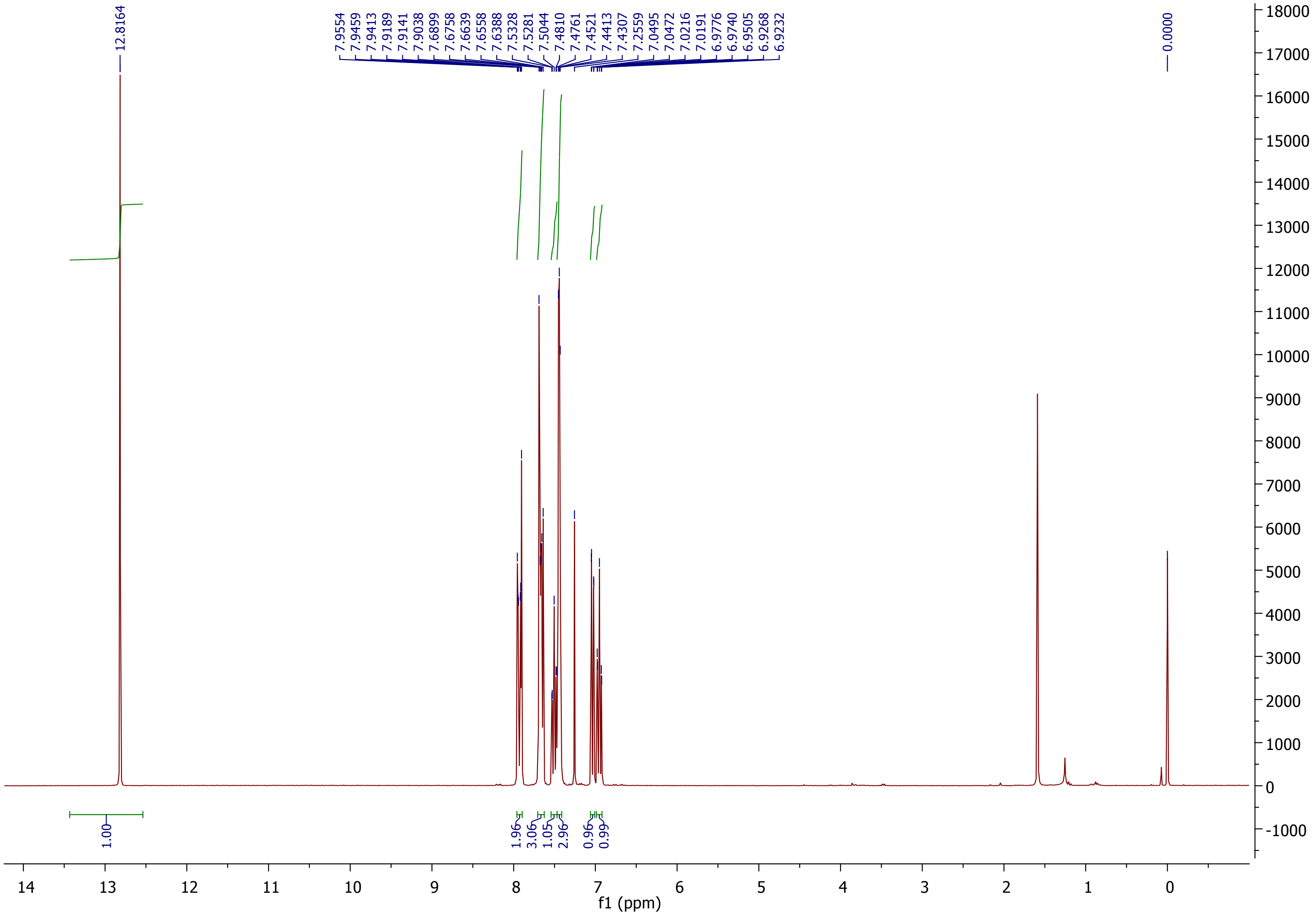

Figure S 16 - ^{1}H NMR Spectra of Compound **19** (δ, $CDCl_3$, 500 MHz)

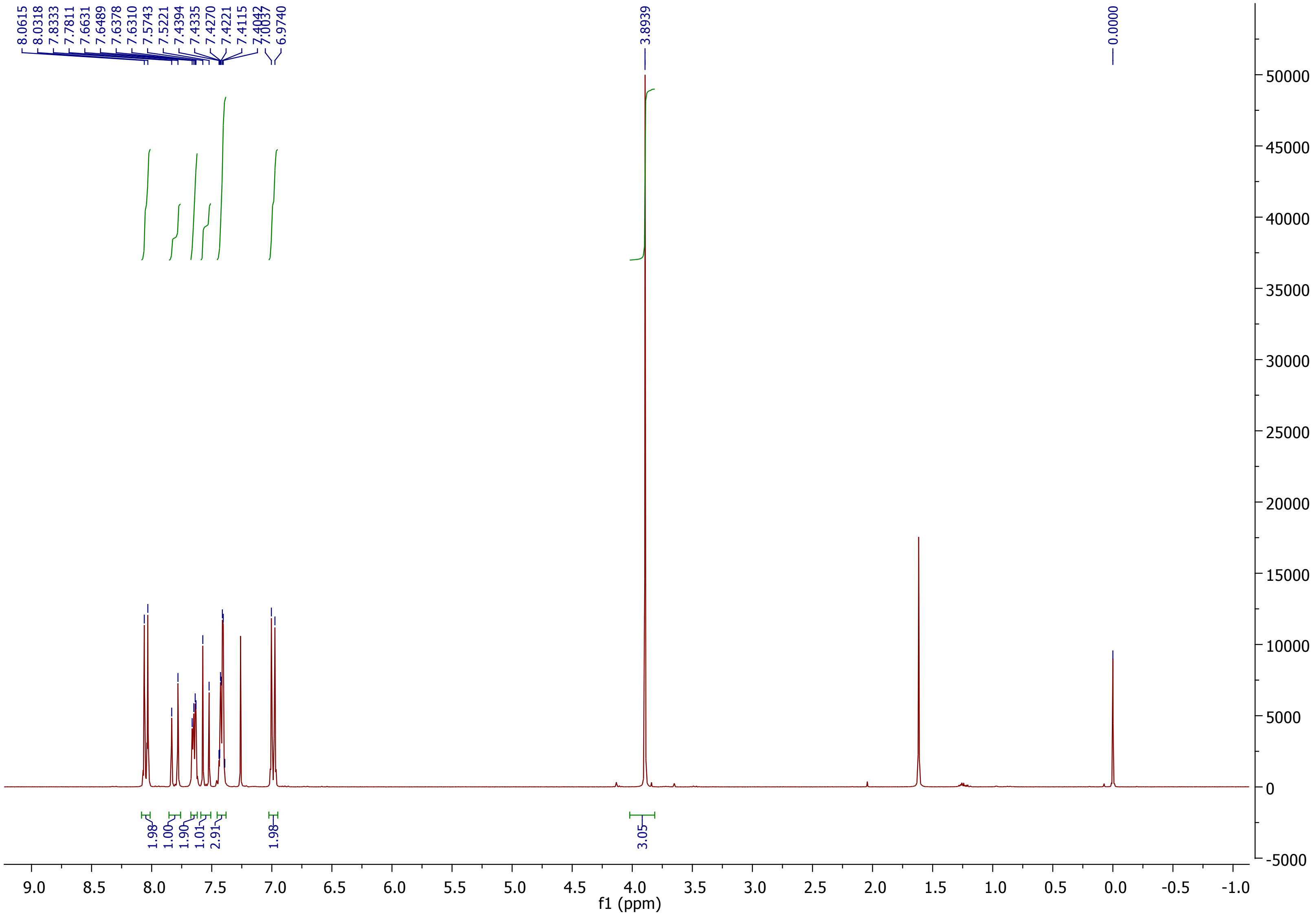

Figure S 17 - ^{1}H NMR Spectra of Compound **20** (δ, $CDCl_3$, 500 MHz)

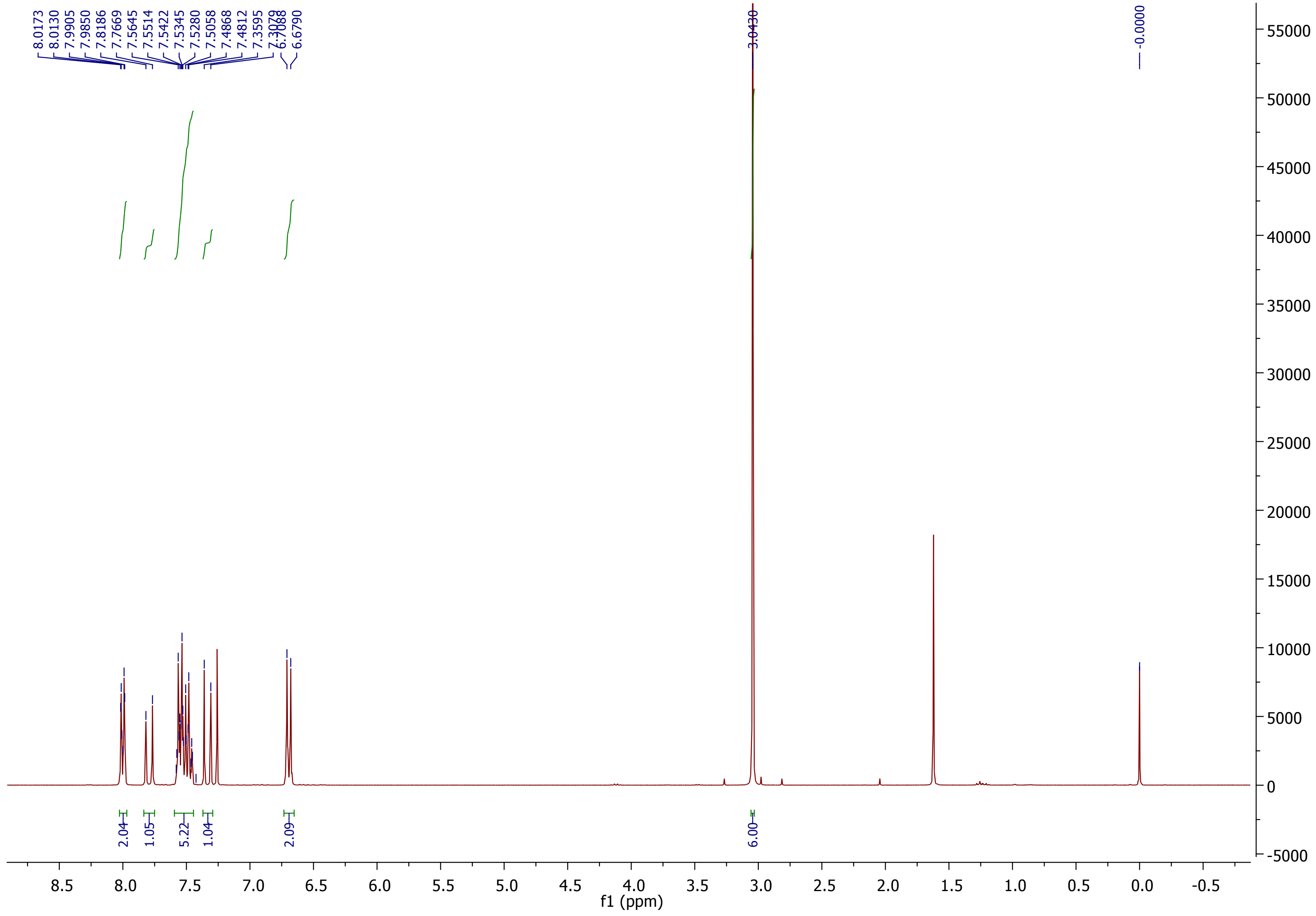

Figure S 18 - ^{1}H NMR Spectrum of Compound **21** (δ, $CDCl_3$, 500 MHz)

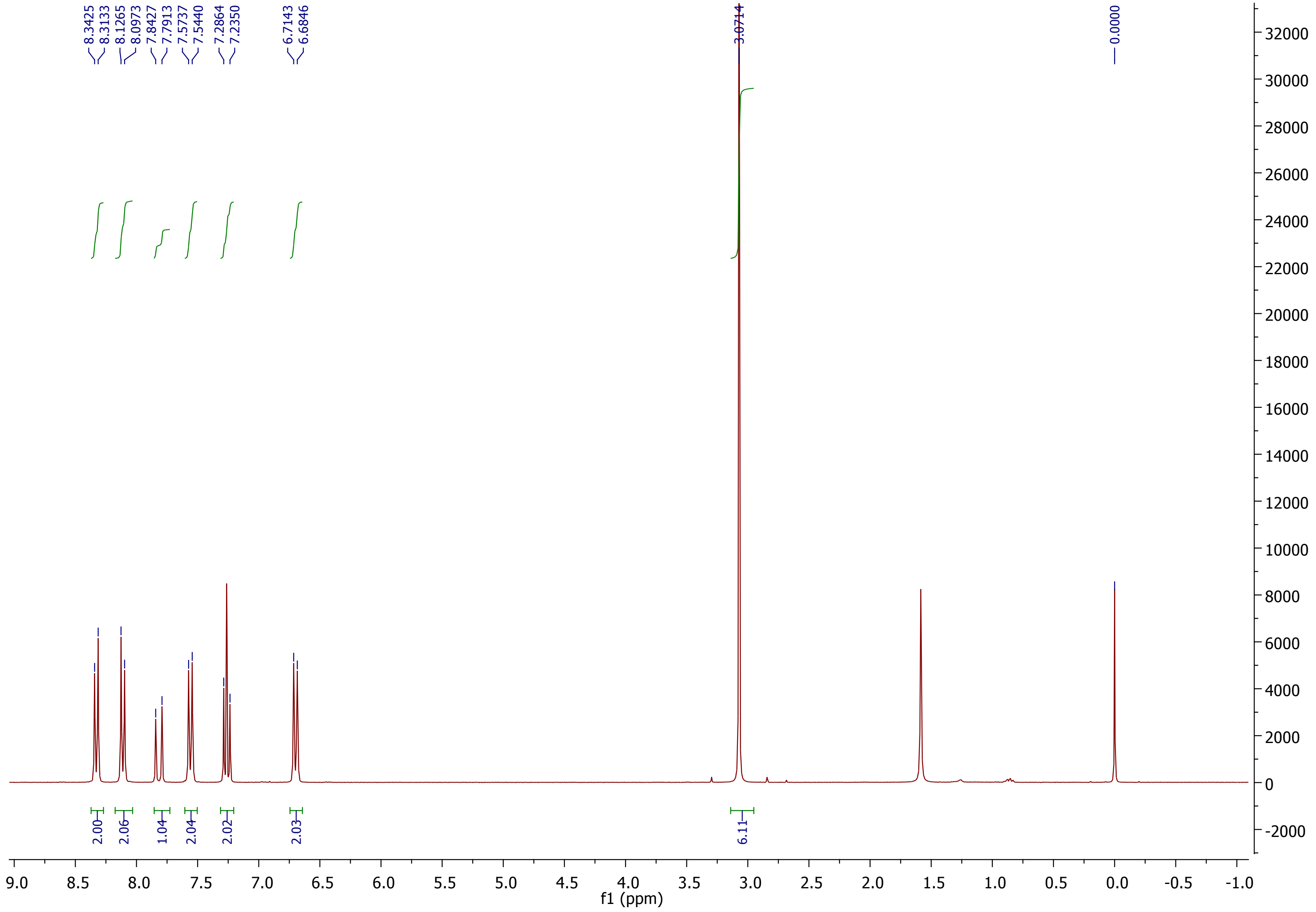

Figure S 19 - ^{1}H NMR Spectrum of Compound **22** (δ, $CDCl_3$, 500 MHz)

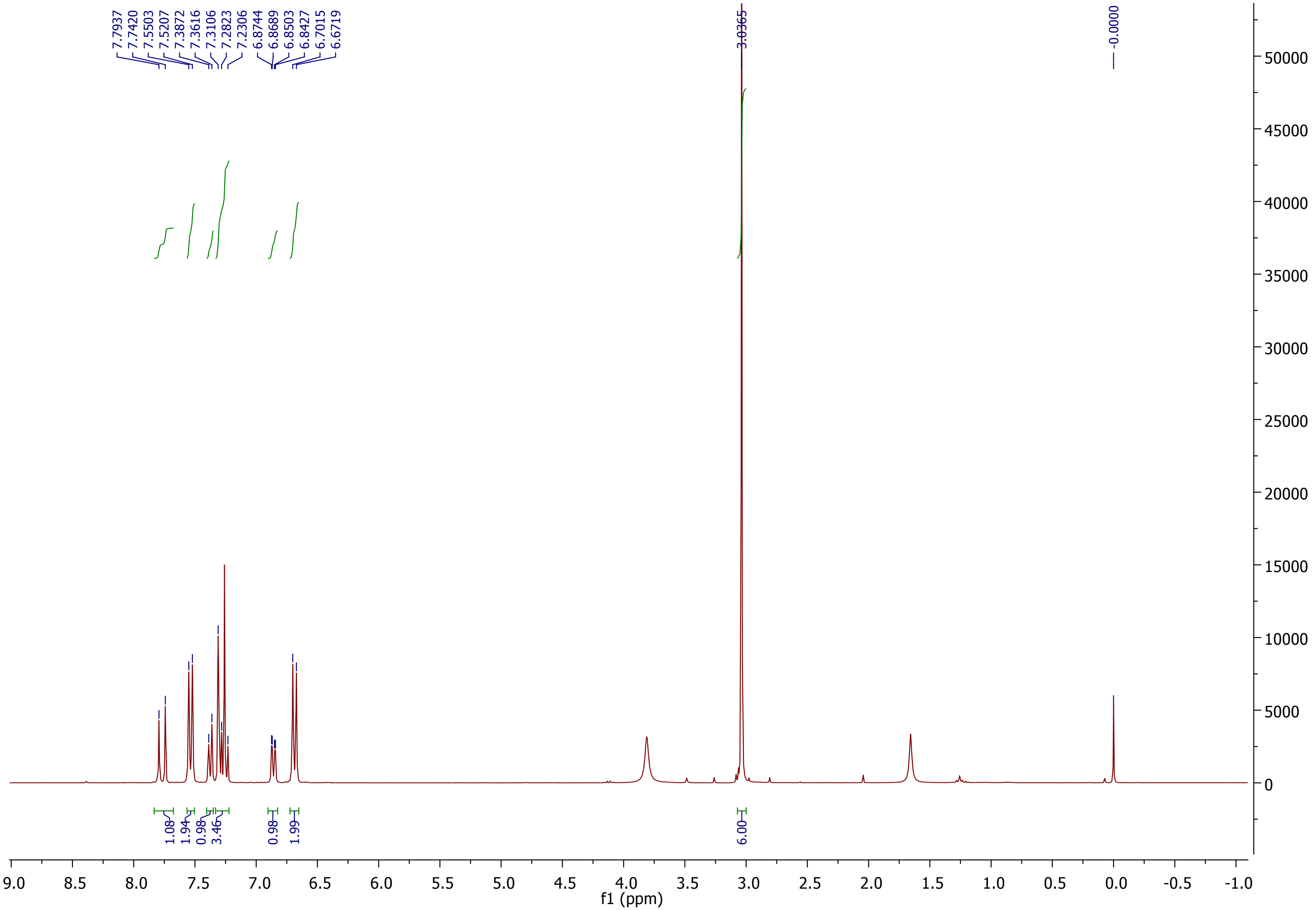

Figure S 20 - ^{1}H NMR Spectrum of Compound **23** (δ, $CDCl_3$, 500 MHz)

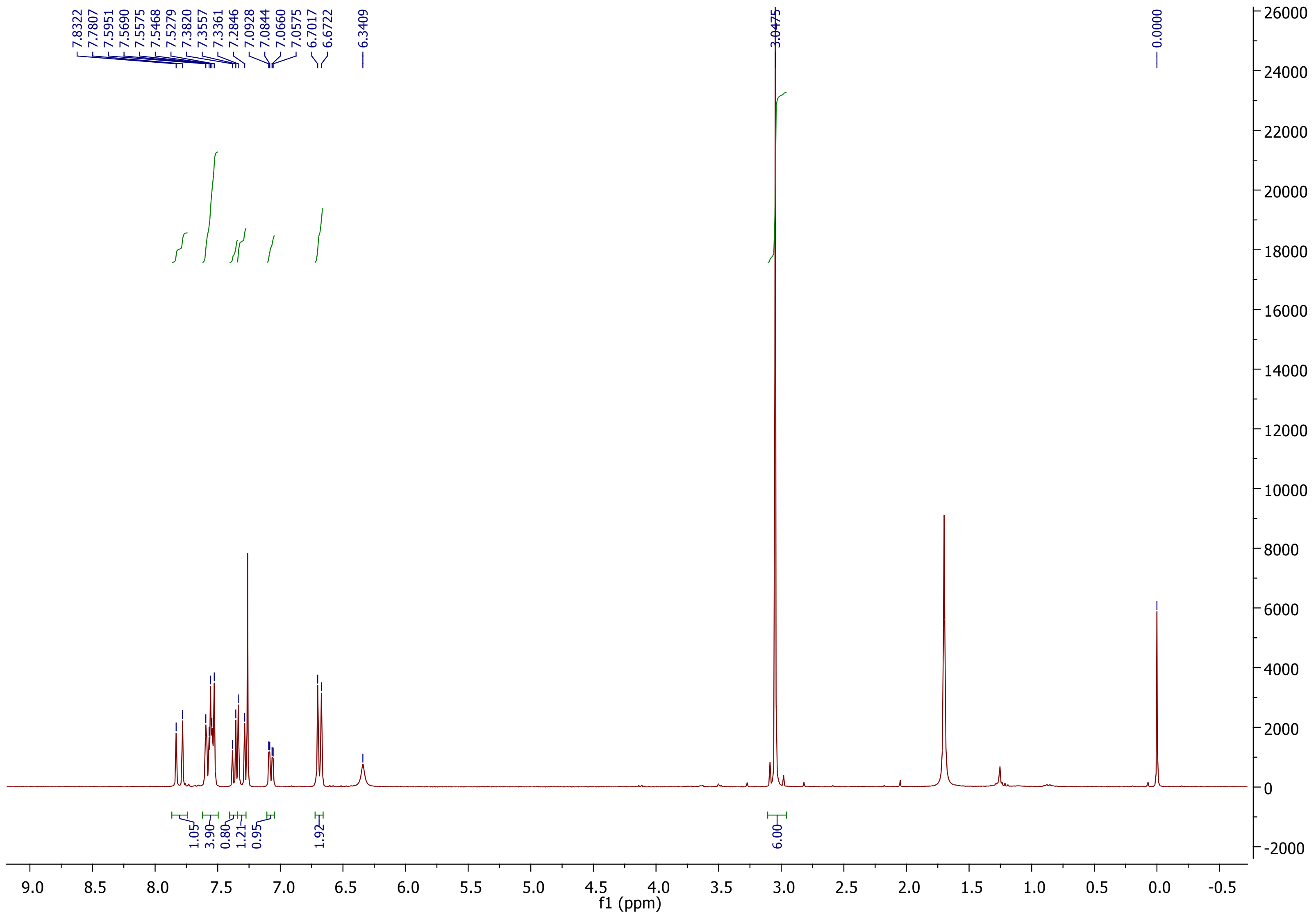

Figure S 21 - ^{1}H NMR Spectrum of Compound **24** (δ, $CDCl_3$, 500 MHz)

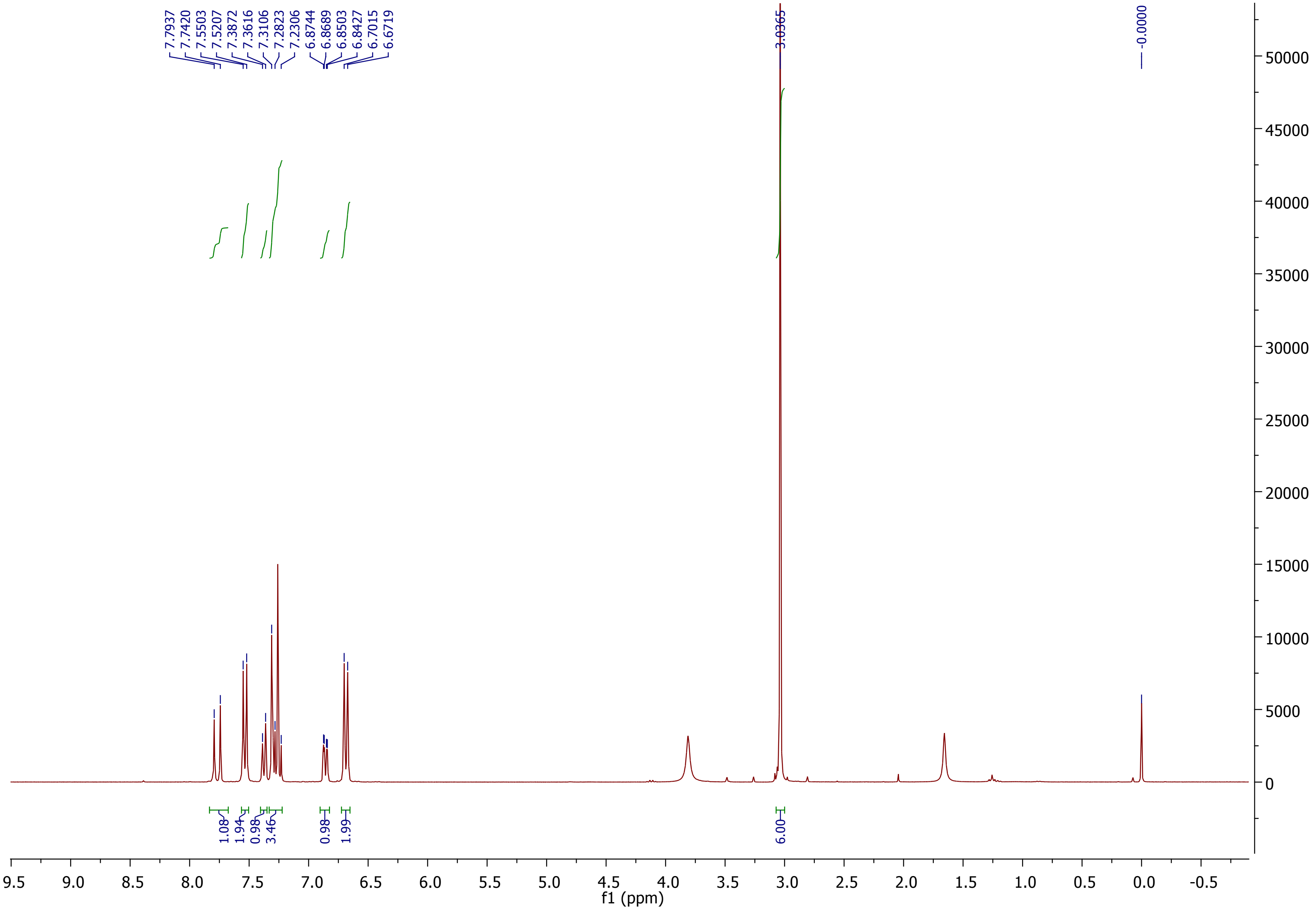

Figure S 22 - ^{1}H NMR Spectrum of Compound **25** (δ, $CDCl_3$, 500 MHz)

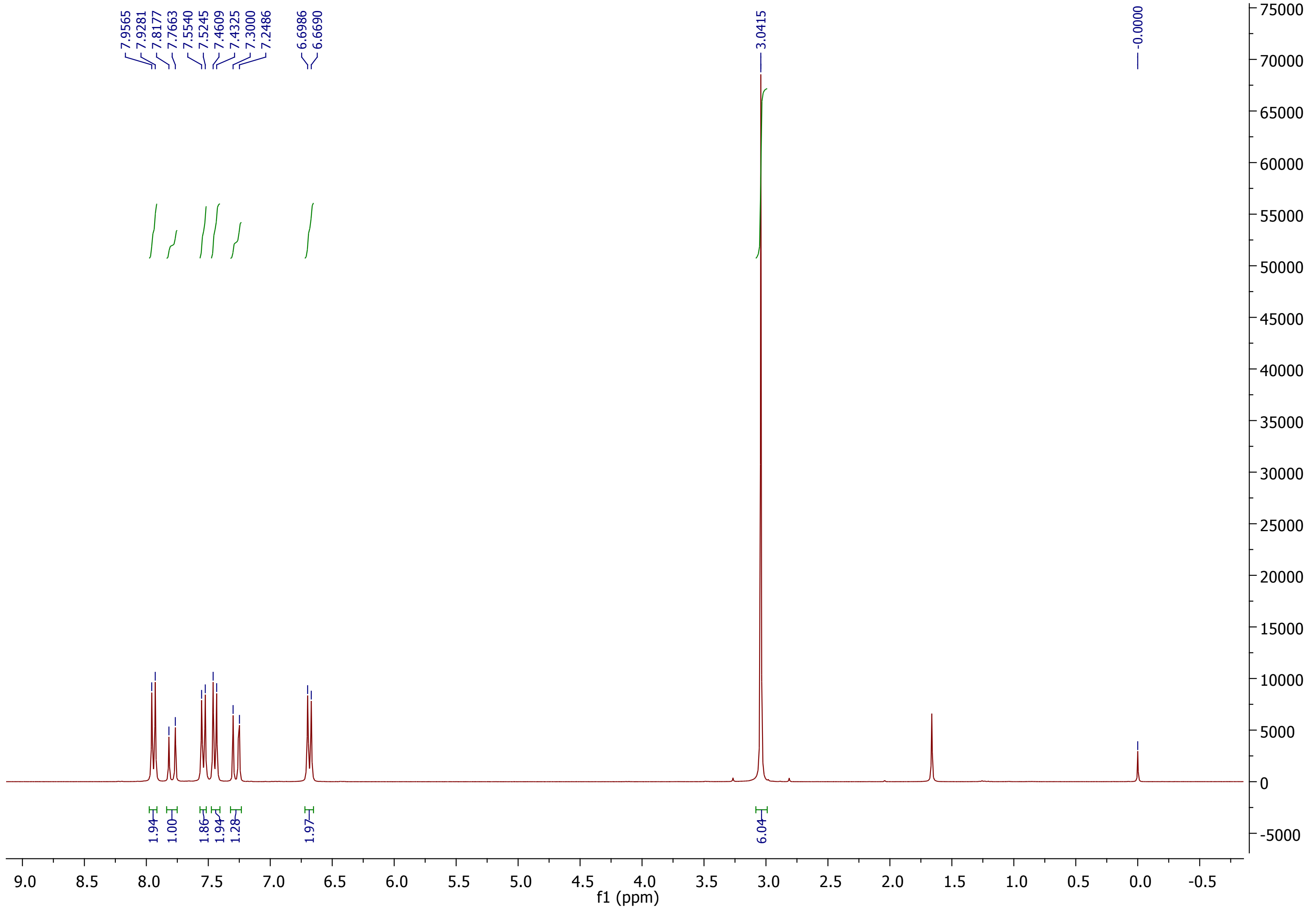

Figure S 23 - ^{1}H NMR Spectrum of Compound **26** (δ, $CDCl_3$, 500 MHz)

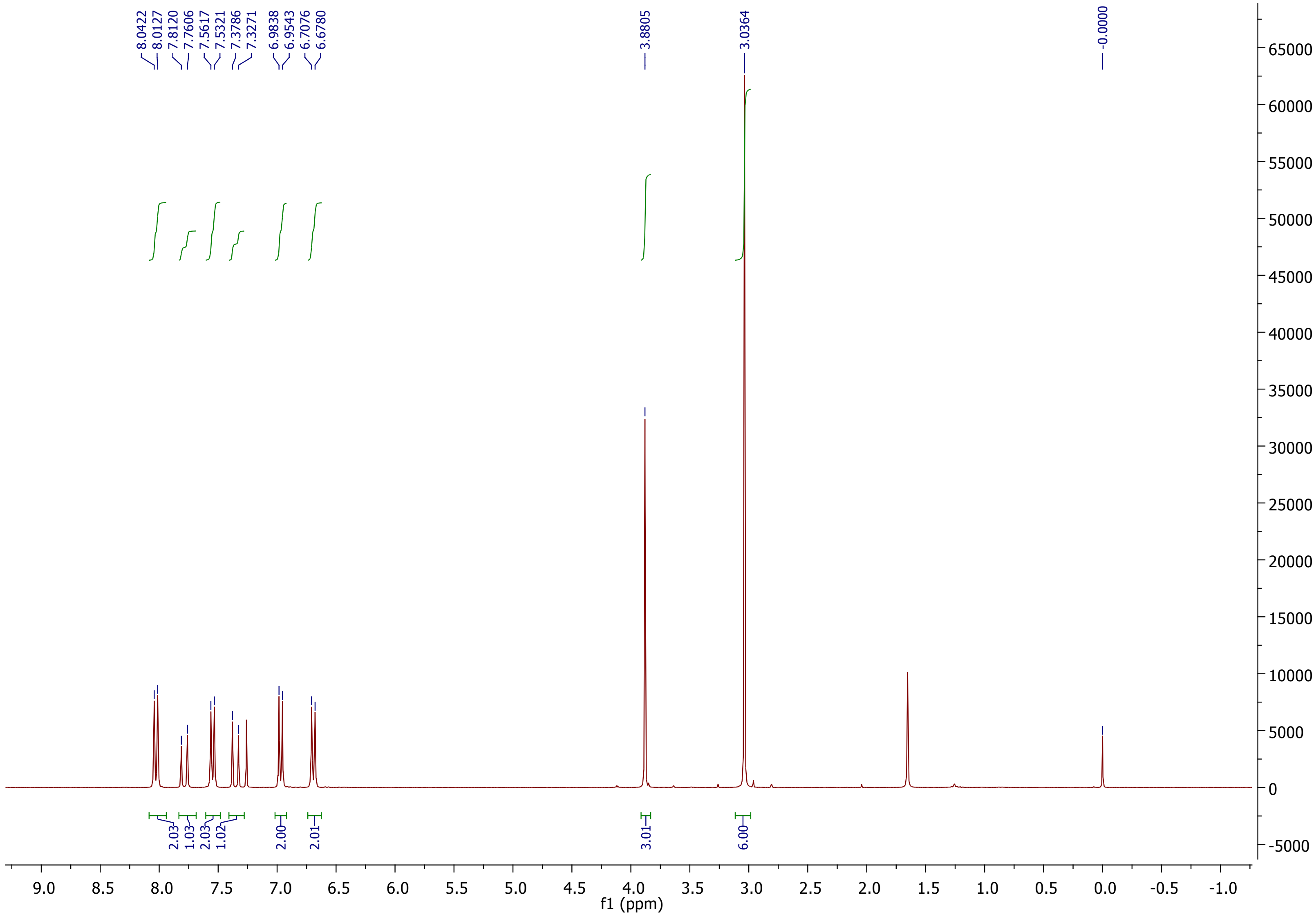

Figure S 24 - ^{1}H NMR Spectrum of Compound **27** (δ, $CDCl_3$, 500 MHz)

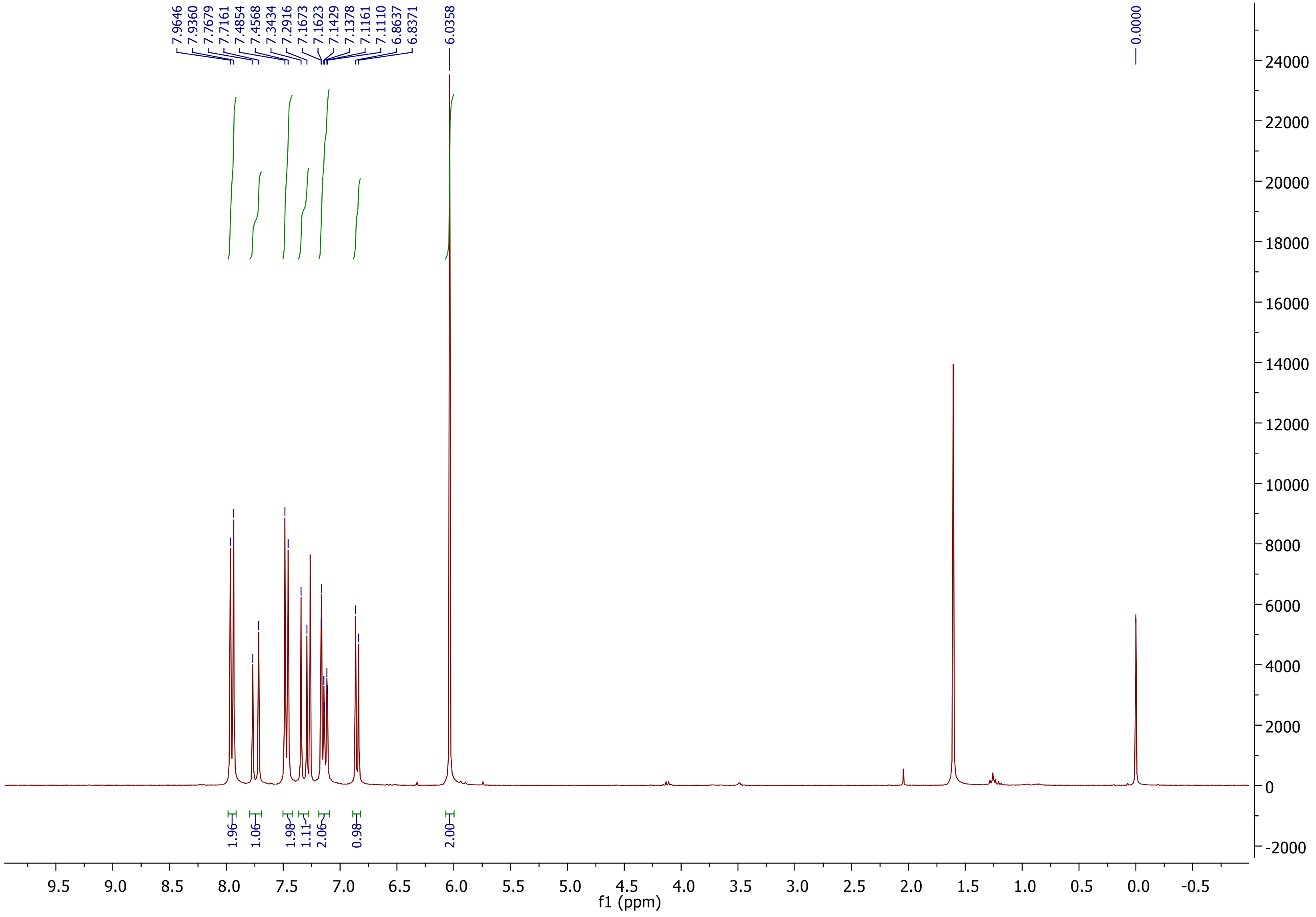

Figure S 25 - ^{1}H NMR Spectrum of Compound **28** (δ, $CDCl_3$, 500 MHz)

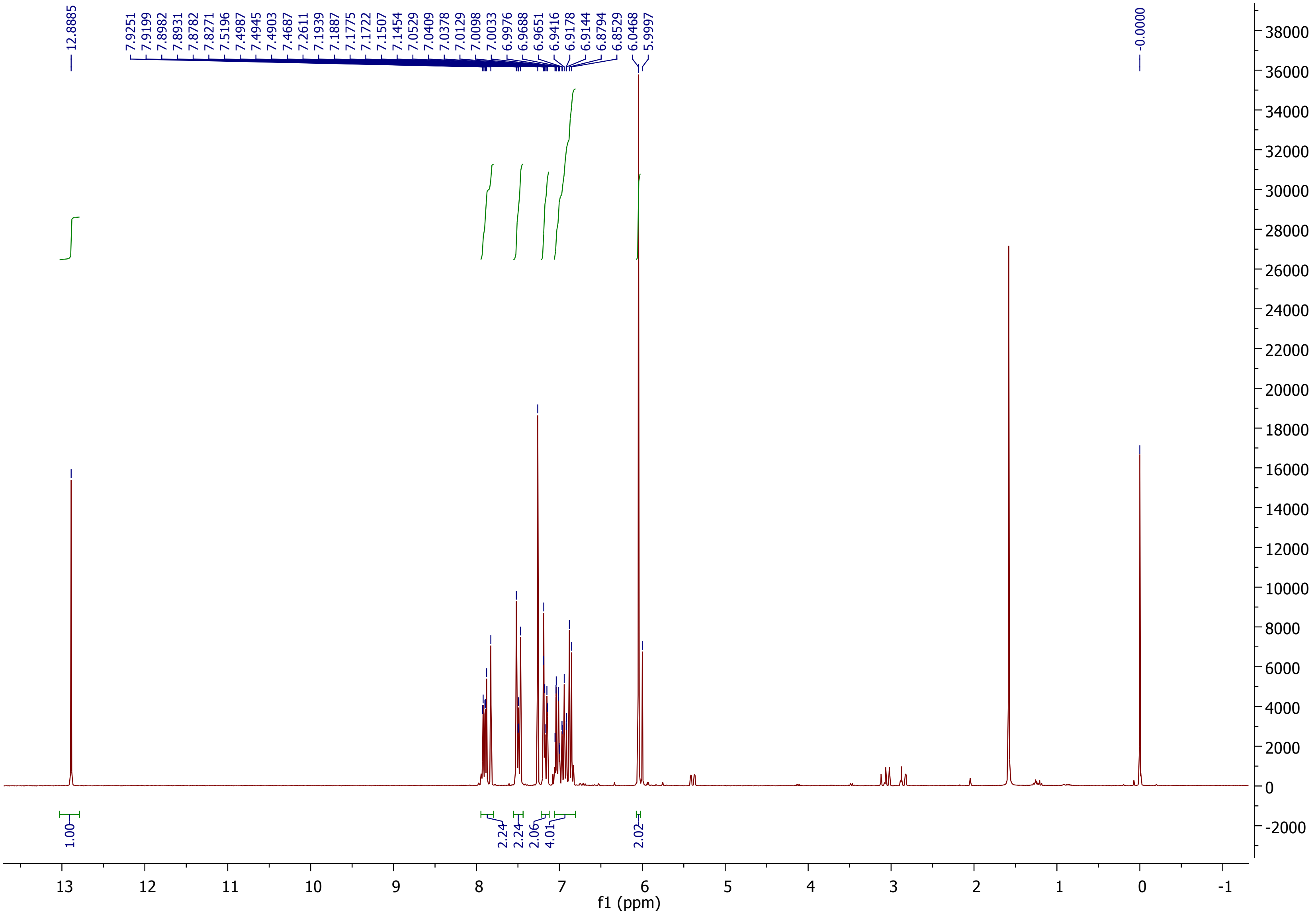

Figure S 26 - ^{1}H NMR Spectrum of Compound **29** (δ, $CDCl_3$, 500 MHz)

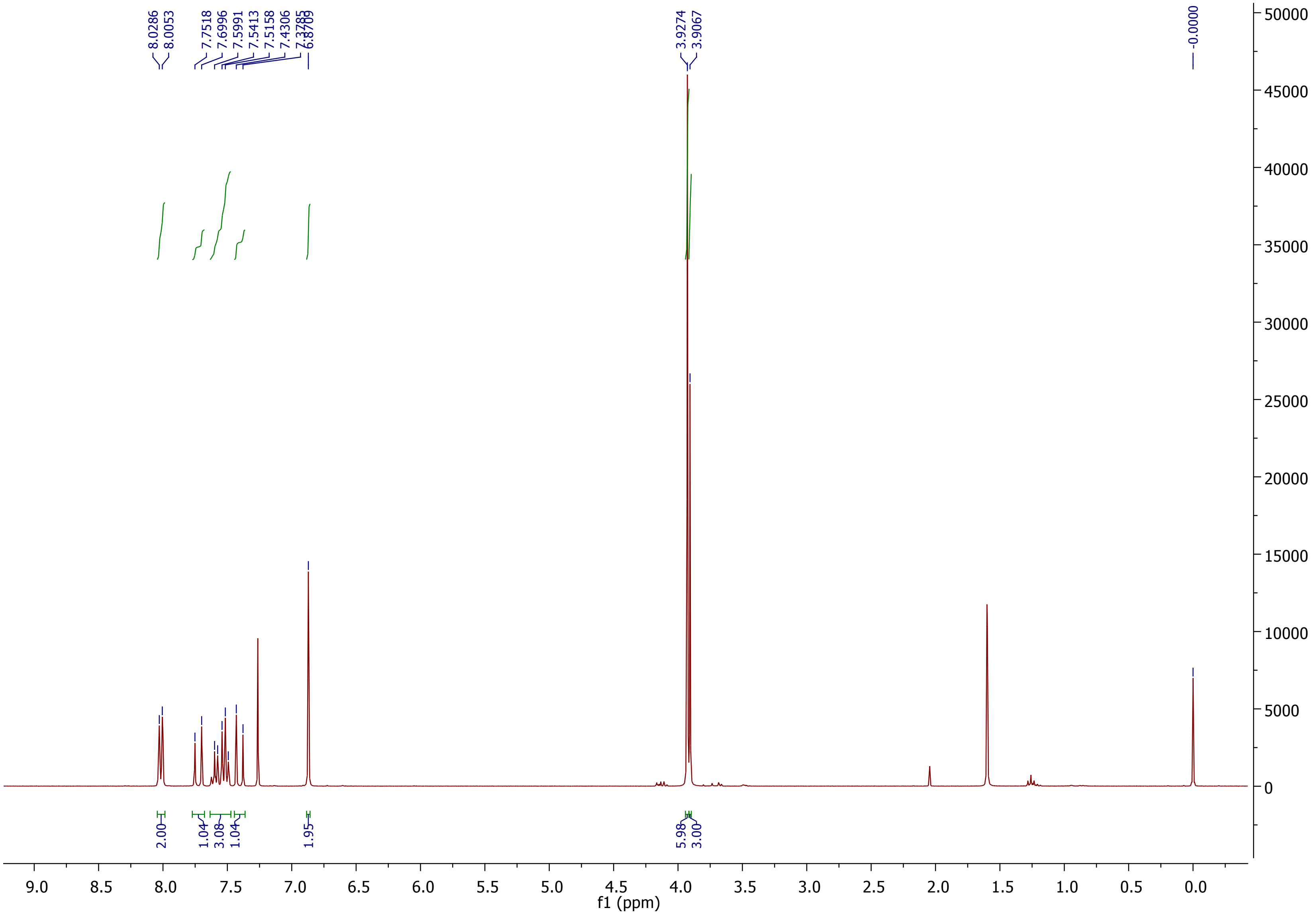

Figure S 27 - ^{1}H NMR Spectrum of Compound **30** (δ, $CDCl_3$, 500 MHz)

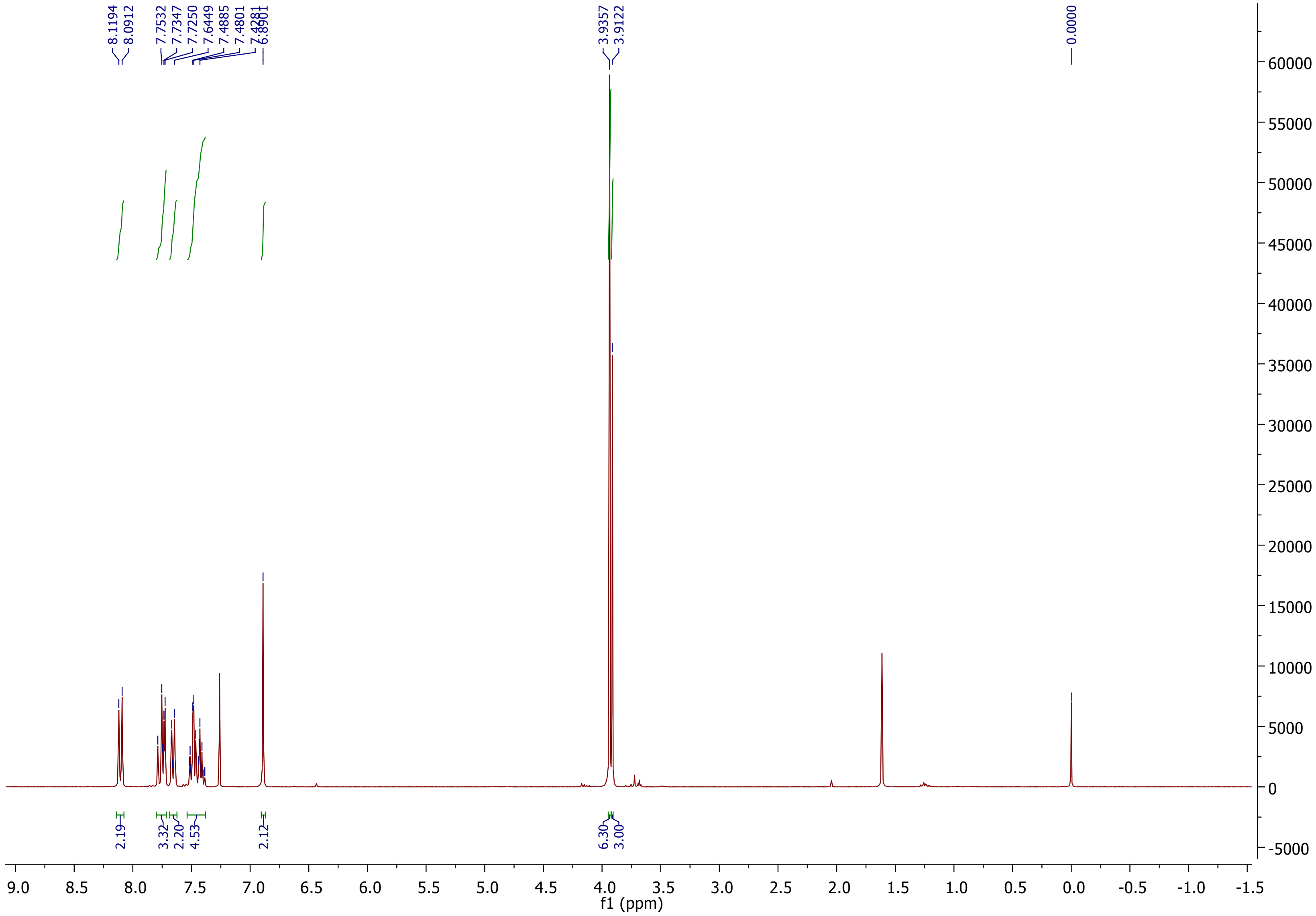

Figure S 28 - ^{1}H NMR Spectrum of Compound **31** (δ, $CDCl_3$, 500 MHz)

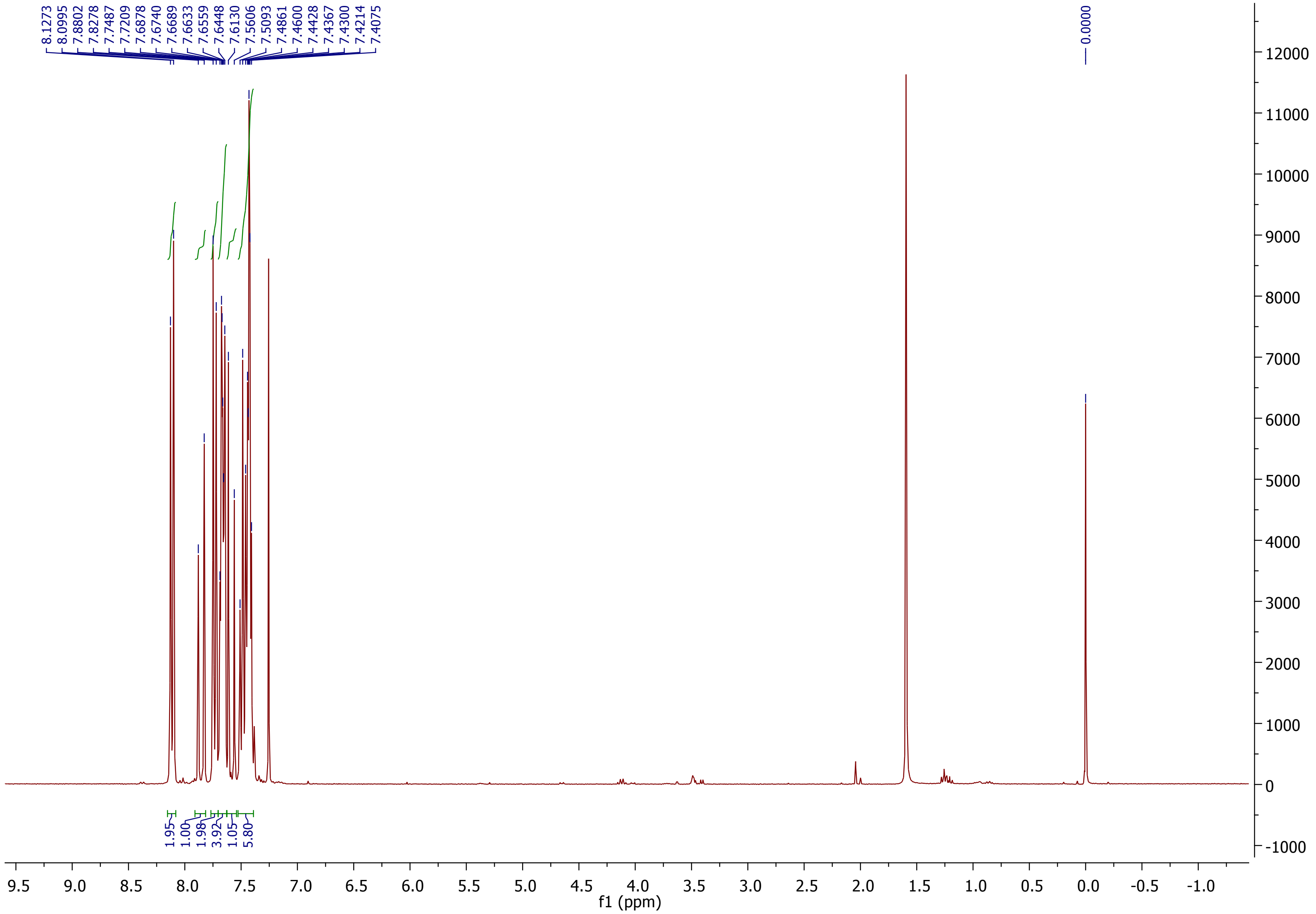

Figure S 29 - ^{1}H NMR Spectrum of Compound **32** (δ, $CDCl_3$, 500 MHz)

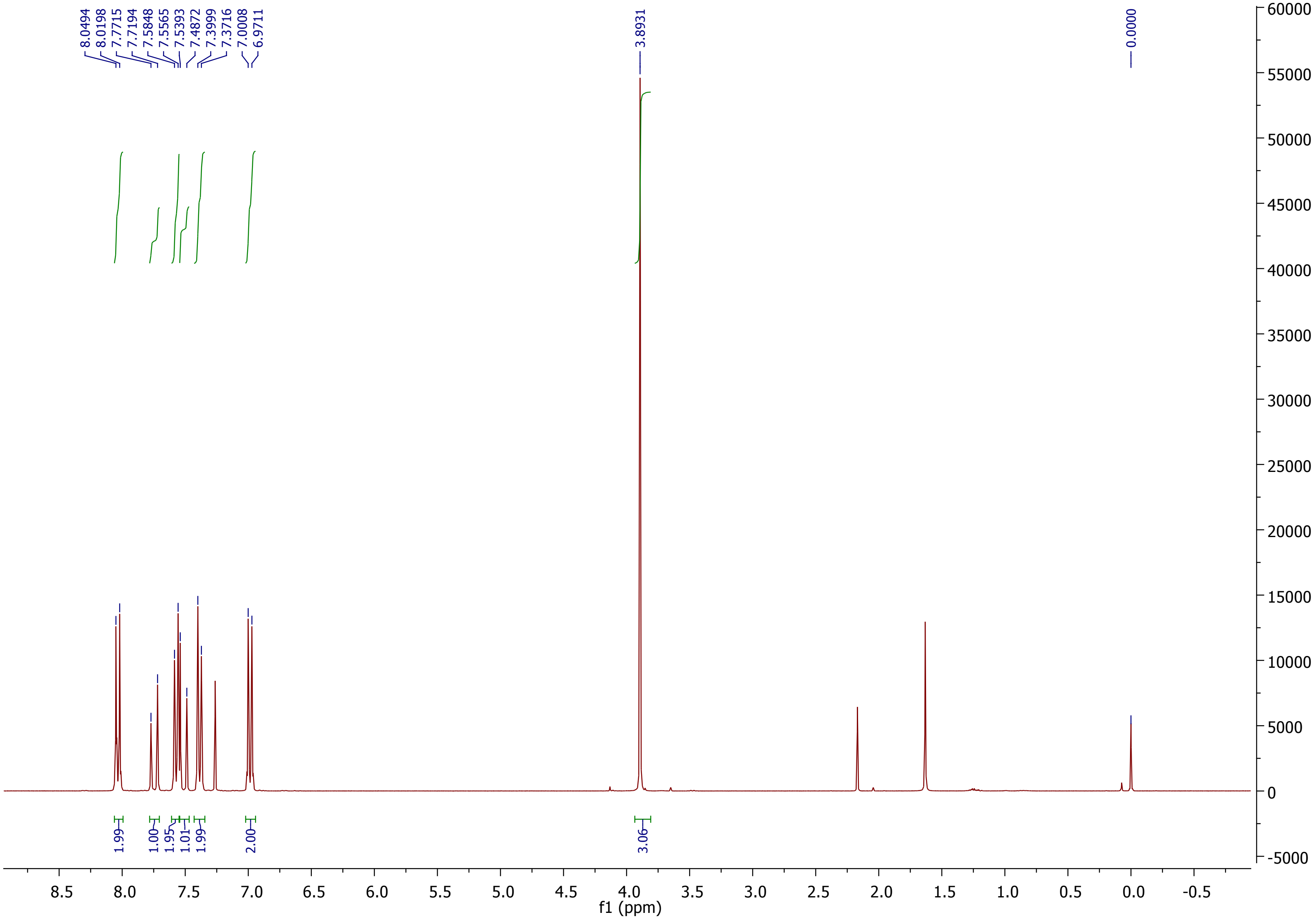

Figure S 30 - ^{1}H NMR Spectrum of Compound **33** (δ, $CDCl_3$, 500 MHz)

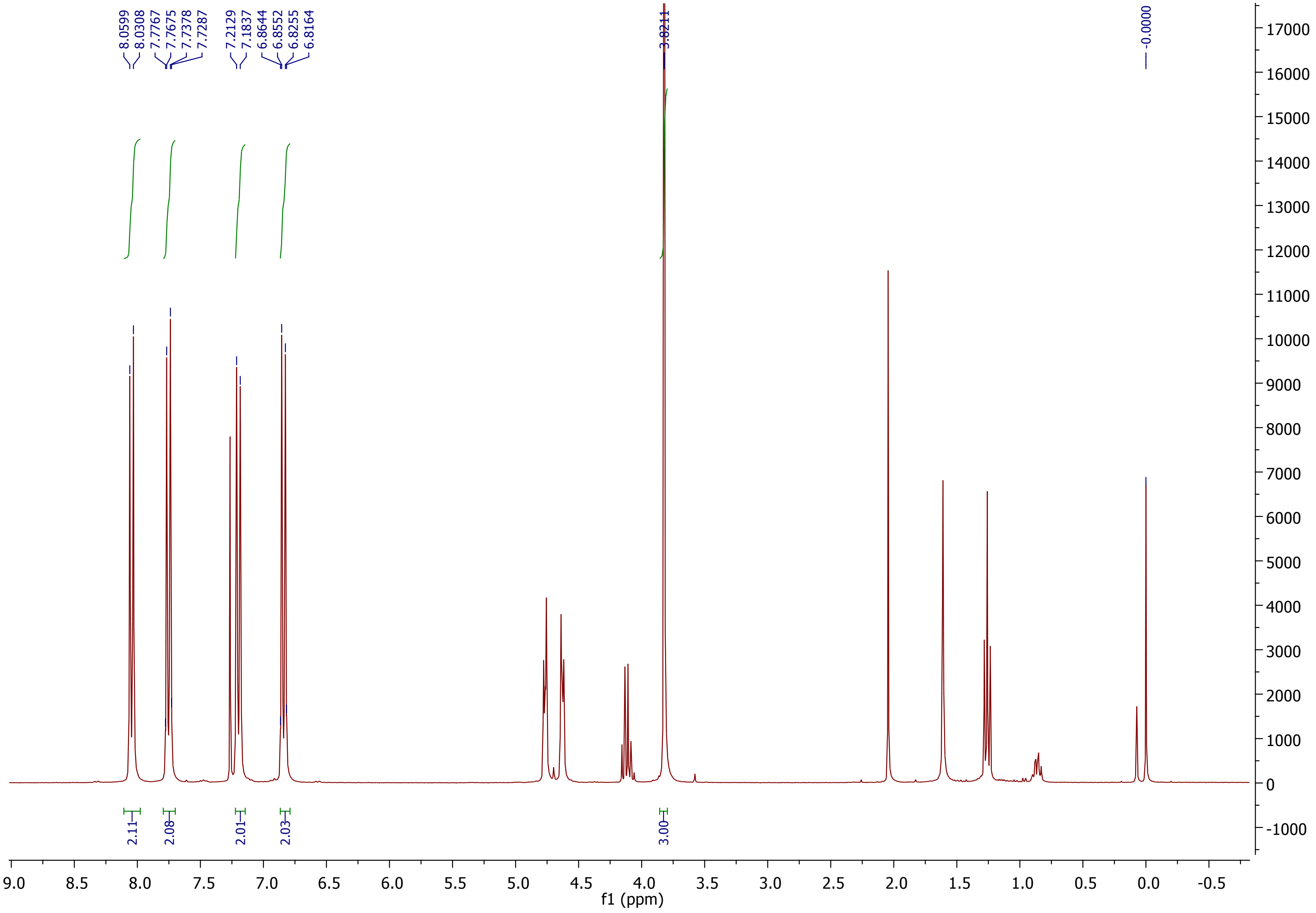

Figure S 31 - ^{1}H NMR Spectrum of Compound **34** (δ, $CDCl_3$, 500 MHz)

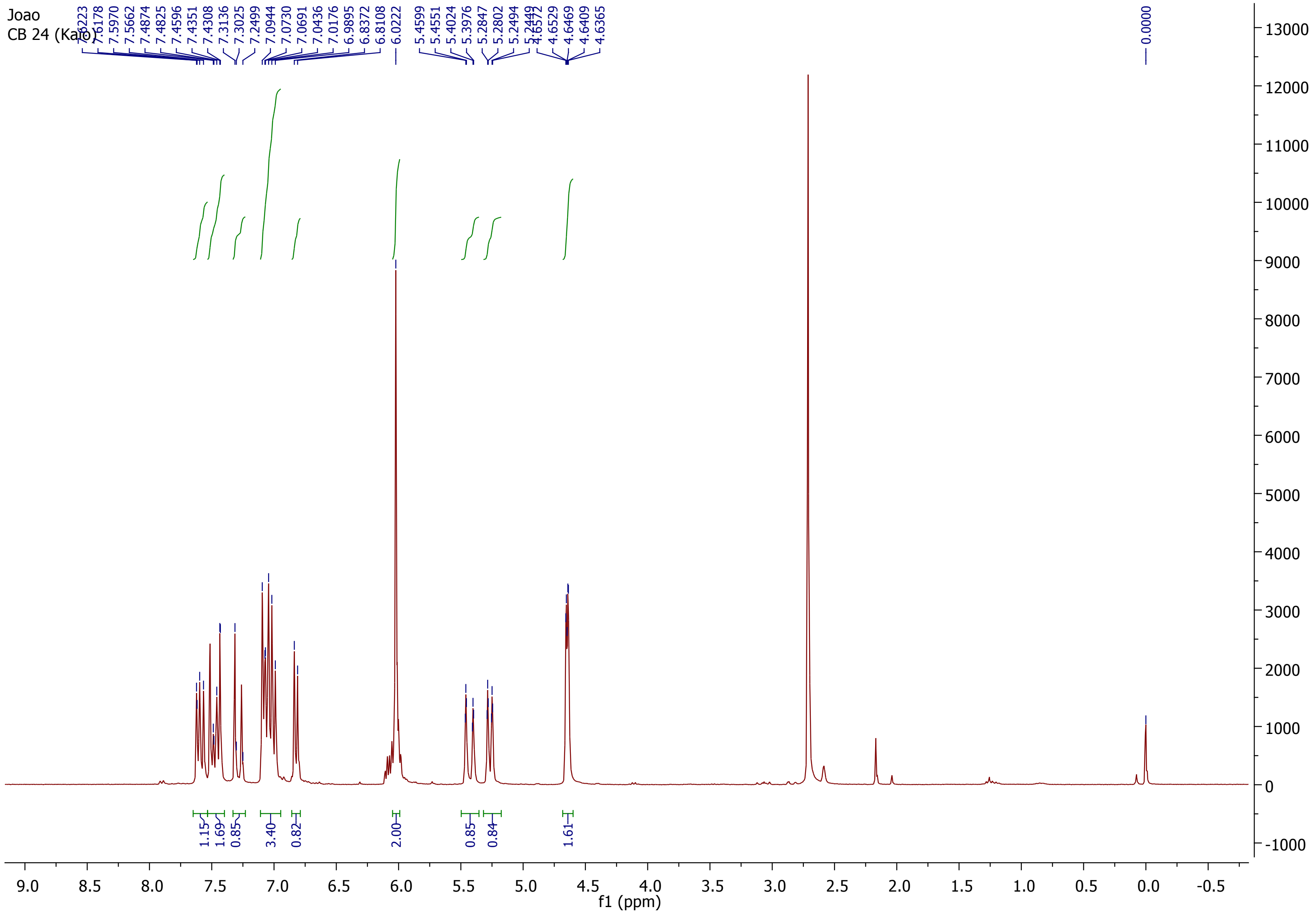

Figure S 32 - ^{1}H NMR Spectrum of Compound **35** (δ, $CDCl_3$, 500 MHz)

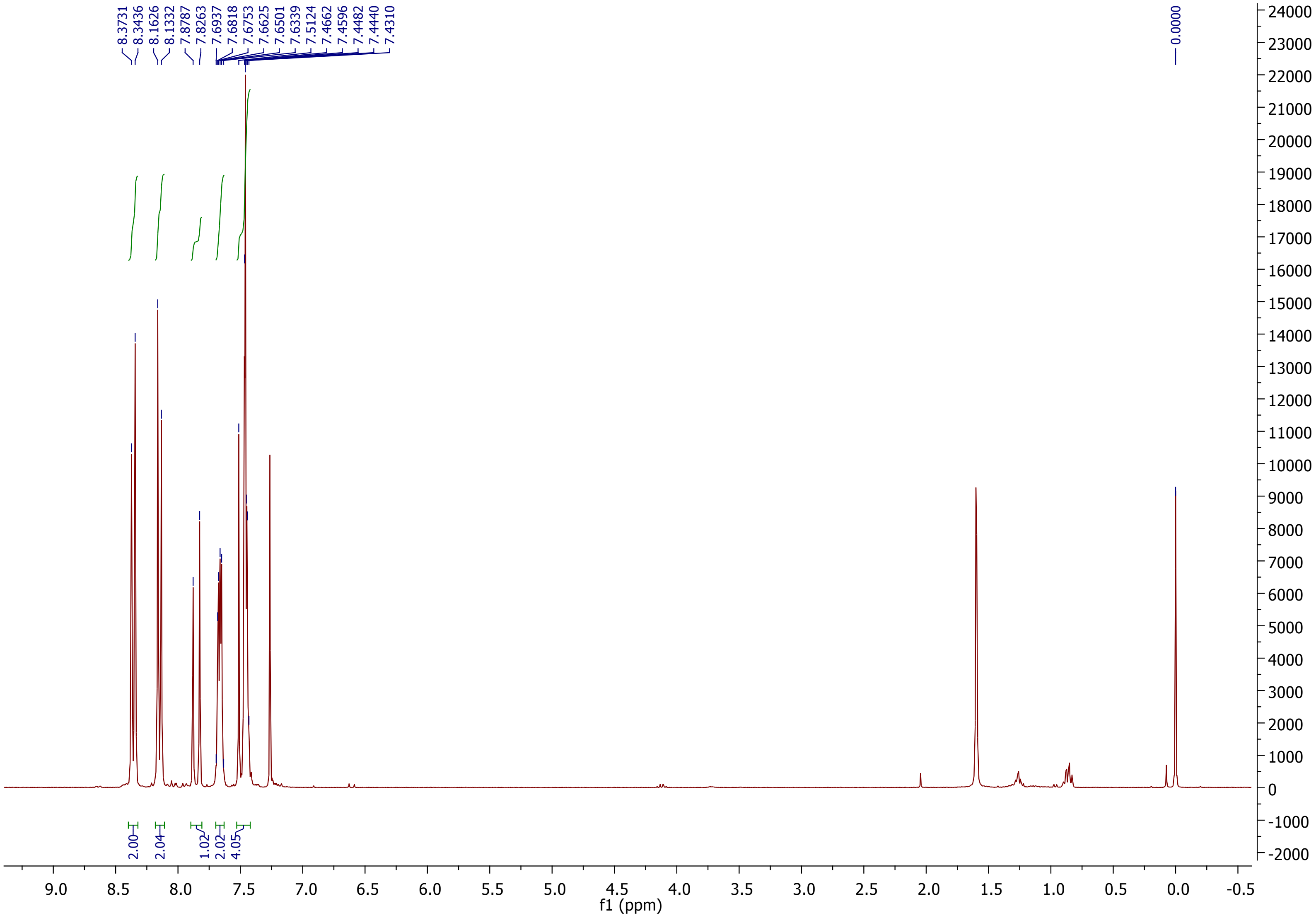

Figure S 33 - ^{1}H NMR Spectrum of Compound **36** (δ, $CDCl_3$, 500 MHz)

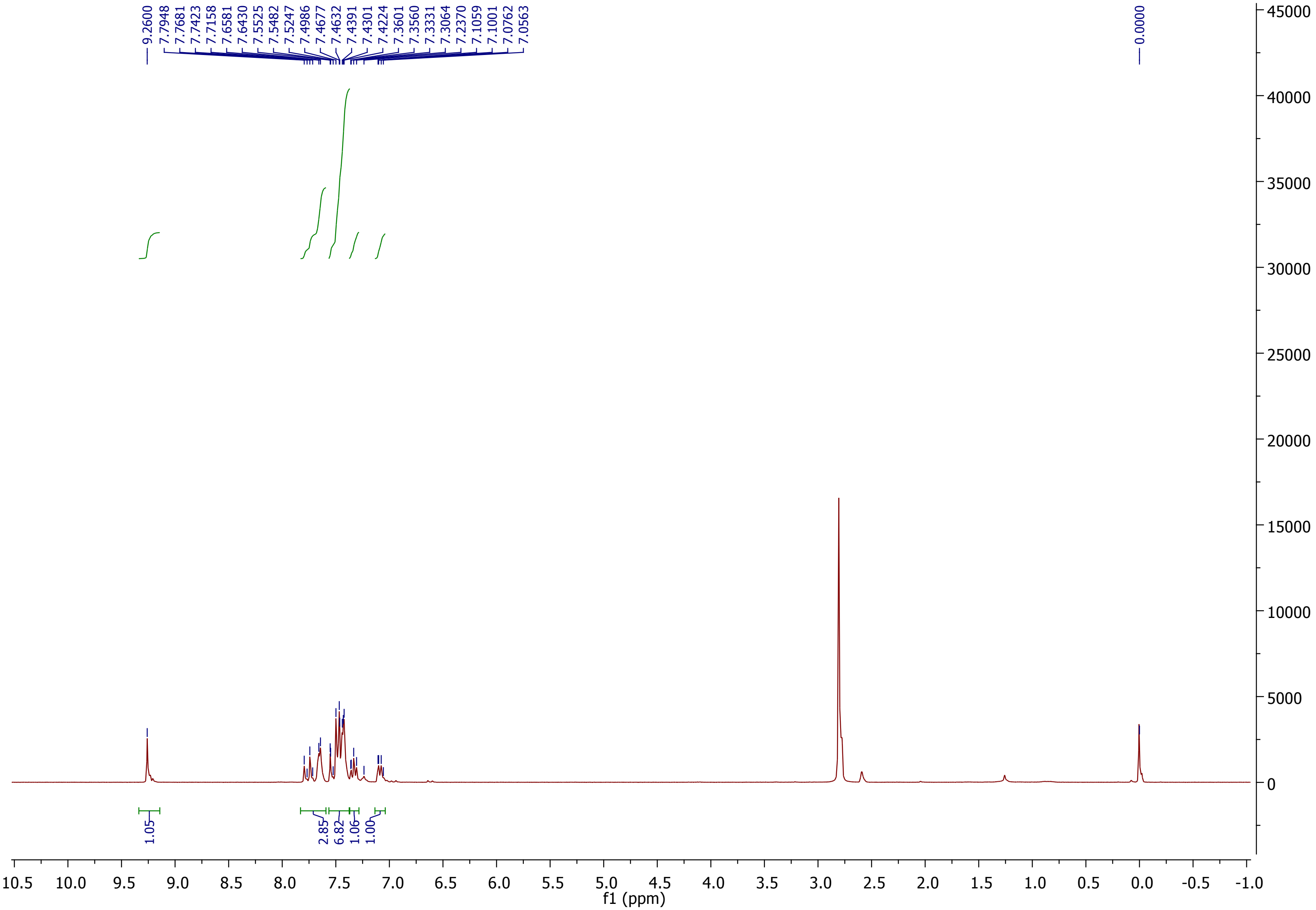

Figure S 34 - ^{1}H NMR Spectrum of Compound **37** (δ, $CDCl_3$, 500 MHz)
